# Y chromosomal noncoding RNAs regulate autosomal gene expression via piRNAs in mouse testis

**DOI:** 10.1186/s12915-021-01125-x

**Published:** 2021-09-09

**Authors:** Hemakumar M. Reddy, Rupa Bhattacharya, Shrish Tiwari, Kankadeb Mishra, Pranatharthi Annapurna, Zeenath Jehan, Nissankararao Mary Praveena, Jomini Liza Alex, Vishnu M. Dhople, Lalji Singh, Mahadevan Sivaramakrishnan, Anurag Chaturvedi, Nandini Rangaraj, Thomas Michael Shiju, Badanapuram Sreedevi, Sachin Kumar, Ram Reddy Dereddi, Sunayana M. Rayabandla, Rachel A. Jesudasan

**Affiliations:** 1 Centre for Cellular and Molecular Biology (CCMB), Uppal Road, Hyderabad, Telangana 500007 India; 2 Present address: Brown University BioMed Division, Department of Molecular Biology, Cell Biology and Biochemistry, 185 Meeting Street room 257, Sidney Frank Life Sciences Building, Providence, RI 02912 USA; 3 Pennington, NJ 08534 USA; 4 Department of Cell Biology, Memorial Sloan Kettering Cancer Centre, Rockefeller Research Laboratory, 430 East 67th Street, RRL 445, New York, NY 10065 USA; 5 Departments of Orthopaedic Surgery & Bioengineering, University of Pennsylvania, 376A Stemmler Hall, 36th Street & Hamilton Walk, Philadelphia, PA 19104 USA; 6 Department of Genetics and Molecular Medicines, Vasavi Medical and Research Centre, 6-1-91 Khairatabad, Hyderabad, 500 004 India; 7 Department of Functional Genomics, Ernst-Moritz-Arndt-University of Greifswald Interfaculty Institute for Genetics and Functional Genomics, Friedrich-Ludwig-Jahn-Straße 15 a, 17487 Greifswald, Germany; 8 Jubilant Biosystems Ltd., #96, Industrial Suburb, 2nd Stage, Yeshwantpur, Bangalore, Karnataka 560022 India; 9 Environmental Genomics Group, School of Biosciences, University of Birmingham, Birmingham, UK; 10 Lerner Research Institute, Cleveland Clinic, Cleveland, Ohio 44120 USA; 11 Institute for Anatomy and Cell Biology, building-307, Heidelberg, Germany; 12 Telangana Social Welfare Residential Degree College for Women, Suryapet, Telangana 508213 India; 13 Department of Genetics, Osmania University, Hyderabad, Telangana 500007 India; 14 Inter University Centre for Genomics & Gene Technology, Karyavattom Campus, University of Kerala, Trivandrum, Kerala India

**Keywords:** Mouse Y chromosome, Long noncoding RNA, Alternative splicing, piRNA, *Pirmy*, *Pirmy*-like RNAs, Male sterility, Comparative sperm proteomics, Autosomal gene regulation

## Abstract

**Background:**

Deciphering the functions of Y chromosome in mammals has been slow owing to the presence of repeats. Some of these repeats transcribe coding RNAs, the roles of which have been studied. Functions of the noncoding transcripts from Y chromosomal repeats however, remain unclear. While a majority of the genes expressed during spermatogenesis are autosomal, mice with different deletions of the long arm of the Y chromosome (Yq) were previously also shown to be characterized by subfertility, sterility and sperm abnormalities, suggesting the presence of effectors of spermatogenesis at this location. Here we report a set of novel noncoding RNAs from mouse Yq and explore their connection to some of the autosomal genes expressed in testis.

**Results:**

We describe a set of novel mouse male-specific Y long arm (MSYq)-derived long noncoding (lnc) transcripts, named *Pirmy* and *Pirmy*-like RNAs. *Pirmy* shows a large number of splice variants in testis. We also identified *Pirmy*-like RNAs present in multiple copies at different loci on mouse Y chromosome. Further, we identified eight differentially expressed autosome-encoded sperm proteins in a mutant mouse strain, XY^RIII^qdel (2/3 Yq-deleted). *Pirmy* and *Pirmy*-like RNAs have homology to 5′/3′UTRs of these deregulated autosomal genes. Several lines of experiments show that these short homologous stretches correspond to piRNAs. Thus, *Pirmy* and *Pirmy*-like RNAs act as templates for several piRNAs. In vitro functional assays reveal putative roles for these piRNAs in regulating autosomal genes.

**Conclusions:**

Our study elucidates a set of autosomal genes that are potentially regulated by MSYq-derived piRNAs in mouse testis. Sperm phenotypes from the Yq-deleted mice seem to be similar to that reported in inter-specific male-sterile hybrids. Taken together, this study provides novel insights into possible role of MSYq-derived ncRNAs in male sterility and speciation.

**Supplementary Information:**

The online version contains supplementary material available at 10.1186/s12915-021-01125-x.

## Background

Y chromosome has come a long way from a single-gene male-determining chromosome to one that houses a few protein-coding genes besides sequences crucial for spermatogenesis and fertility [1–5]. Earlier studies have shown that genes involved in sex determination and spermatogenesis are present on the short arm [6–8]. Several lines of evidence indicate that the male-specific region on long arm of the Y chromosome (MSYq) in mouse is replete with highly repetitive mouse-specific sequences that are expressed in spermatids [9–12]. Previously published data have described two different strains of mice with partial deletions of the long arm of Y chromosome (Yq) [2, 13]. Mice from both the genetic backgrounds exhibit male-sterile phenotypes such as subfertility, sex ratio skewed towards females, reduced number of motile sperms, aberrant sperm motility and sperm head morphological abnormalities [2, 14]. Mice with partial deletions of Yq show sperm abnormalities with less severe phenotype whereas mice with total deletion of the Yq have extensive sperm morphological aberrations and are sterile [15]. This suggested the presence of multicopy spermiogenesis gene(s) on mouse Yq [2, 10, 16]. Subsequently multicopy transcripts such as Y353/B, spermiogenesis-specific transcript on the Y *(Ssty)* and Sycp3-like, Y-linked *(Sly)* from mouse Yq were projected as putative candidate genes for male sterility and spermiogenesis in mice [2, 15, 17–19]. In total, 126 copies of *Sly* and 306 copies of *Ssty* have been reported from mouse Y chromosome [10]. As mice with partial deletions of Yq (XY^RIII^qdel, 2/3^rd^ interstitial deletion of Yq) show reduced expression of *Ssty* and impaired fertility, this gene (present on Yq) was implicated in spermatogenesis [15]. The next major gene to be discovered on mouse Yq was the multicopy *Sly*. As SLY interacts with a histone acetyl transferase and is an acrosomal protein, the authors suggested that *Sly* could control transcription and acrosome functions [20]. Further, Cocquet and colleagues observed major problems in sperm differentiation when they disrupted functions of *Sly* gene by transgenic delivery of siRNA to the gene [18]. Therefore, *Sly* was conjectured as a putative candidate gene for spermiogenesis [17]. However, subsequently Ward and colleagues showed rescue of *Sly* does not restore the phenotype completely; hence, it was concluded that *Sly* expression alone is not sufficient for spermiogenesis [21]. The SSTY protein appears to be essential for enabling the entry of SLY into the nucleus [22, 23]. We postulated the possibility of yet undiscovered genes in the region involved in male fertility.

Vast majority of the genes required for spermatogenesis and spermiogenesis are non-Y-linked [24–26]. Deletions of the Y chromosome leading to different degrees of male infertility prompted us to also hypothesize interactions between Y-derived transcripts and autosomal genes in male fertility, based on earlier studies in the lab on human Y chromosome [27]. We hypothesized more of such interactions between protein-coding genes on autosomes and noncoding RNAs from the Y chromosome. In this context, we studied a mutant mouse, which had a partial deletion of the long arm of mouse Y chromosome, XY^RIII^qdel, [2] to look for novel genes/regulatory elements, if any, in the deleted region.

Previous studies in the lab identified 300–400 copies of a mouse Y chromosome-specific genomic clone, M34 (DQ907163) [28, 29]. There is a reduction in copy number and transcription of M34 in the XY^RIII^qdel mice that exhibit multiple sperm abnormalities. As deletions of Yq show sperm abnormalities, we reasoned that these repeat sequences could have important functional role(s) in the multistep developmental process of sperm production. In order to understand putative functions of this sequence, first of all we identified a transcript corresponding to M34, *Pirmy*, from mouse testis. Subsequent experiments identified multiple splice variants and related transcripts of *Pirmy*. Parallel experiments identified deregulated proteins in the sperm proteome of the XY^RIII^qdel mice. Interestingly, genes corresponding to all these proteins localized to different autosomes. Further, we showed that the UTRs of these genes bear homology to piRNAs derived from *Pirmy* and *Pirmy*-like RNAs. Thus, our results demonstrate for the first time (i) a set of novel noncoding RNAs (*Pirmy* and *Pirmy*-like RNAs) on mouse Y long arm, (ii) large number of splice variants of *Pirmy*, and the generation of piRNAs from these ncRNAs in mouse testis and (iii) their putative role in regulation of autosomal genes involved in male fertility and reproduction.

## Results

### M34 is transcribed in mouse testis

**Fig. 1:**
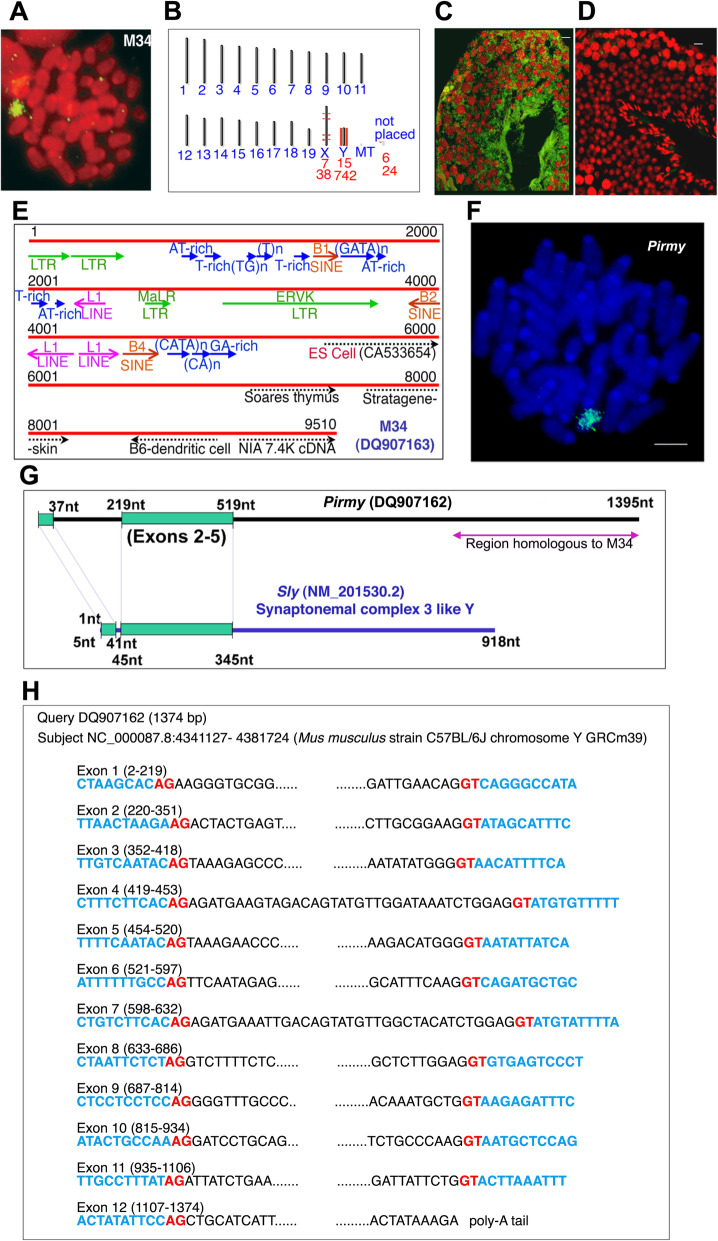
Analysis of M34 (DQ907163) and identification of a novel noncoding RNA. **A** Localization by FISH of the genomic clone, M34 to mouse Y long arm in multiple copies spanning its entire length. **B** Mouse genome map view of M34 BLAST hits (NCBI build 38.1), showing Y chromosomal localization further indicating male-specificity of these repeats. **C** Fluorescent in situ hybridization (FISH) using M34 elicits signals in adult mouse testis. Green fluorescence represents signal from M34, nuclei are counterstained with Propidium iodide (red). Yellow indicates co-localization. **D** Hybridization of M34 onto RNase-treated testis sections does not show signals, indicating that the signals in panel **C** are due to the presence of M34-derived RNA **E** Sequence analysis of the 9.5 kb M34 shows presence of incomplete copies of different repeats like long terminal repeats (LTRs), long interspersed nuclear elements (LINEs), short interspersed nuclear elements (SINEs), endogenous retroviral sequences (ERVK) and simple sequence repeats in both direct and reverse orientations in the clone. ESTs matching to M34 are marked as dotted arrows at the 3′ end. ES cell EST (CA533654) was used as the probe to identify *Pirmy*. **F** Y-specific localization of *Pirmy* cDNA clone (DQ907162) on a mouse metaphase spread by FISH, showing the presence of multiple copies. **G** Partial homology between *Sly* and *Pirmy* (DQ907162), indicating identification of a novel cDNA. Homology region is highlighted in green rectangles. Purple arrow shows the homology to M34. **H** The consensus splice signal sequences (AG/GT) at all intron- exon junctions of DQ907162 (see also Data Sheet)

To address the precise function of M34, we confirmed the localization of the sex- and species-specific repeat (M34) on mouse Yq again by fluorescence in situ hybridization (FISH) (Fig. 1A). BLAST analysis of M34 sequence against mouse whole genome also showed maximum similarity to Y chromosome (> 97% identity) with few hits on the X (Fig. 1B*,* NCBI build 38.1). M34 was then analysed for expression in adult mouse testis. FISH using M34 as a probe revealed abundant transcription in testis (Fig. 1C). Pretreatment with RNase abolished these signals confirming the presence of RNA (Fig. 1D). Expression profiling by FISH in testes showed the presence of M34 transcripts in 18.5-day embryos, newborns and 1-month-old mice (30 days postpartum) (Additional file 1: Fig. S1), suggesting transcription of this repeat in mouse testis from early developmental stages.

### Isolation of a novel polyadenylated noncoding transcript from mouse Y chromosome

Sequence analysis of the 9.51 kb M34 clone using Tandem Repeats Finder (TRF) and RepeatMasker identified different simple sequence repeats and partial mid-repetitive sequences like LINEs, SINEs and LTR elements, which constitute ~ 35% of the total M34 sequence (Fig. 1E). A number of gene prediction programmes like GENSCAN, GrailEXP, MZEF and GeneMark did not predict any genes within M34 with consistency. BLAST analysis of M34 sequence against the EST database of NCBI (NCBI build 36.1) identified five ESTs at the 3′ end of M34 sequence (Fig. 1E). Expression of these ESTs was observed in embryos from at least 13.5d onwards (data not shown). Two of these ESTs showed male-specific expression by reverse transcription PCR (RT-PCR) analysis.

In order to identify cDNA(s) corresponding to M34 in testis, one of the male-specific ESTs (CA533654) was used to screen a mouse testis cDNA library. A 1395-nt-long polyadenylated Y-specific cDNA was isolated and this was named *Pirmy*—piRNA from mouse Y chromosome (DQ907162). FISH on to mouse metaphase spreads showed that *Pirmy* is present only on the Y chromosome in multiple copies (Fig. 1F), similar to that of the genomic clone M34. BLAST of *Pirmy* against the nucleotide database of NCBI picked up only mouse sequences with statistically significant alignments (e-value < 4e^−04^). This suggests that *Pirmy* is specific to mouse. BLAST analysis using *Pirmy* against the mouse genome plus transcriptome database showed homology to *Sly* transcript in exons 1–5. The exons 2–5 were identical in *Pirmy* and *Sly* (Fig. 1G). The region of homology is less than a quarter of the length of *Pirmy* and is confined to the 5′ end. This does not include the Cor1 domain of SLY protein, which starts from exon 7 of *Sly* transcript. Exons 11 and 12 of *Pirmy* harbours homology to M34. Thus, M34 has no homology to *Sly*. The entire sequence of *Pirmy* localizes to the Y chromosome at 4341127-4381724 (GRCm39). BLAST search against the nucleotide database identified a reference sequence NR164186, which was annotated as a long noncoding RNA using evidence data for transcript exon combination from *Pirmy*. The exon-intron junctions of *Pirmy* contain consensus splice signal sequences AG/GT (Fig. 1H).

### Splice variants of *Pirmy* in mouse testis

**Fig. 2:**
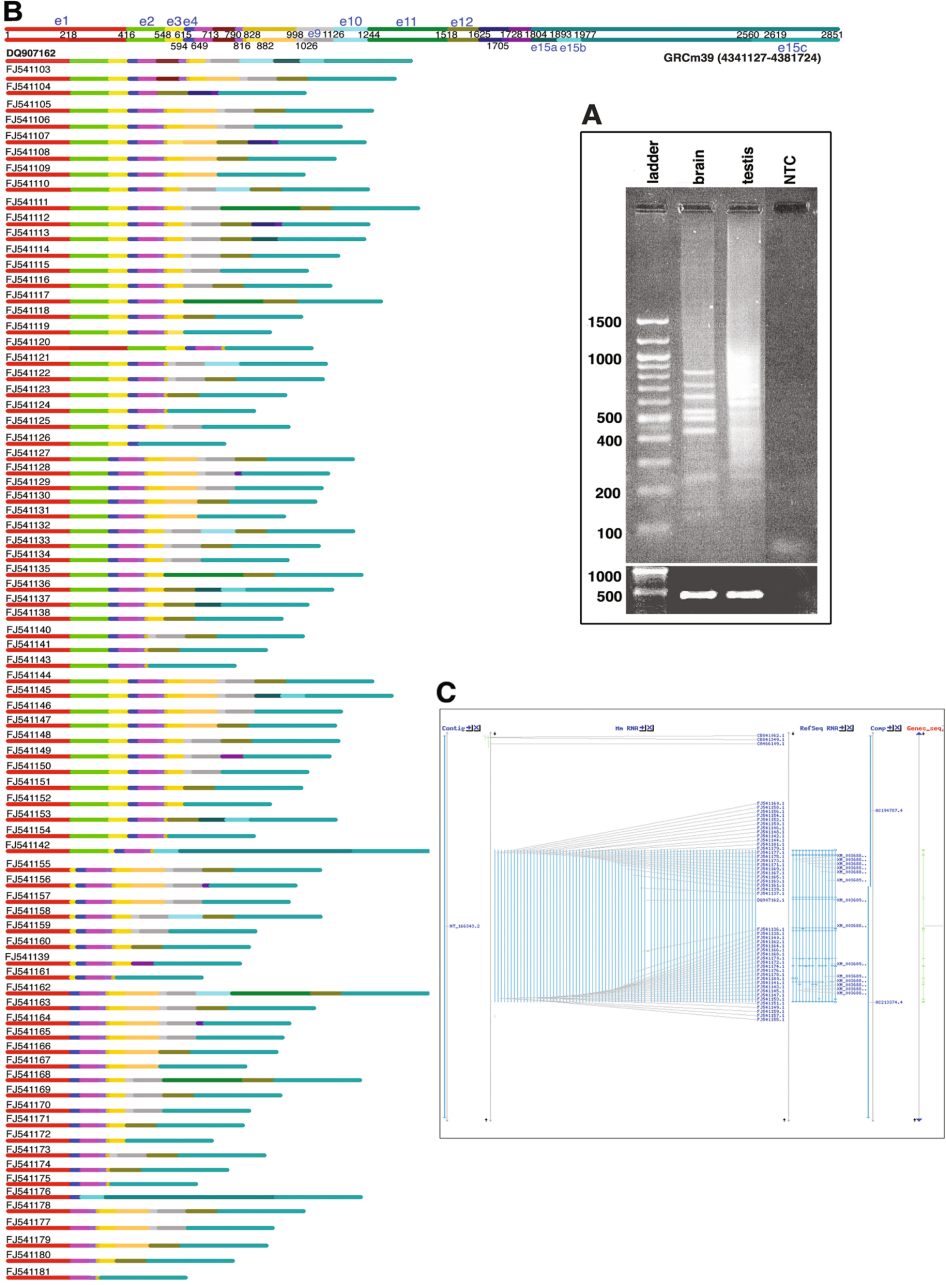
Identification of multiple splice variants of *Pirmy*. **A** RT-PCR amplification of *Pirmy* showed multiple amplicons in both testis and brain. The RT-PCR products from testis were cloned and sequenced to identify the splice variants. NTC is the non-template control. Bottom panel shows *Gapdh* control for checking the integrity and quality of RNA. **B** Colour coded line diagram showing extensive alternative splicing of *Pirmy*. The 80 splice variants (DQ907162, FJ541103-FJ541181) depicted here localize to Y: 4341127-4381724 (GRC m39). Each exon is represented by the same colour in different isoforms. Sizes of the exons are to scale. Top two lines show the representation of all exons present at this locus according to their order in the genomic sequence as e1, e2, etc. Line 2 indicates the nucleotide positions of each exon in a scenario where all the exons are present. The exons have been arranged in linear order. **C** BLAST analysis against mouse genome localizes the splice variants of *Pirmy* to NT_166343.2

**Fig. 3:**
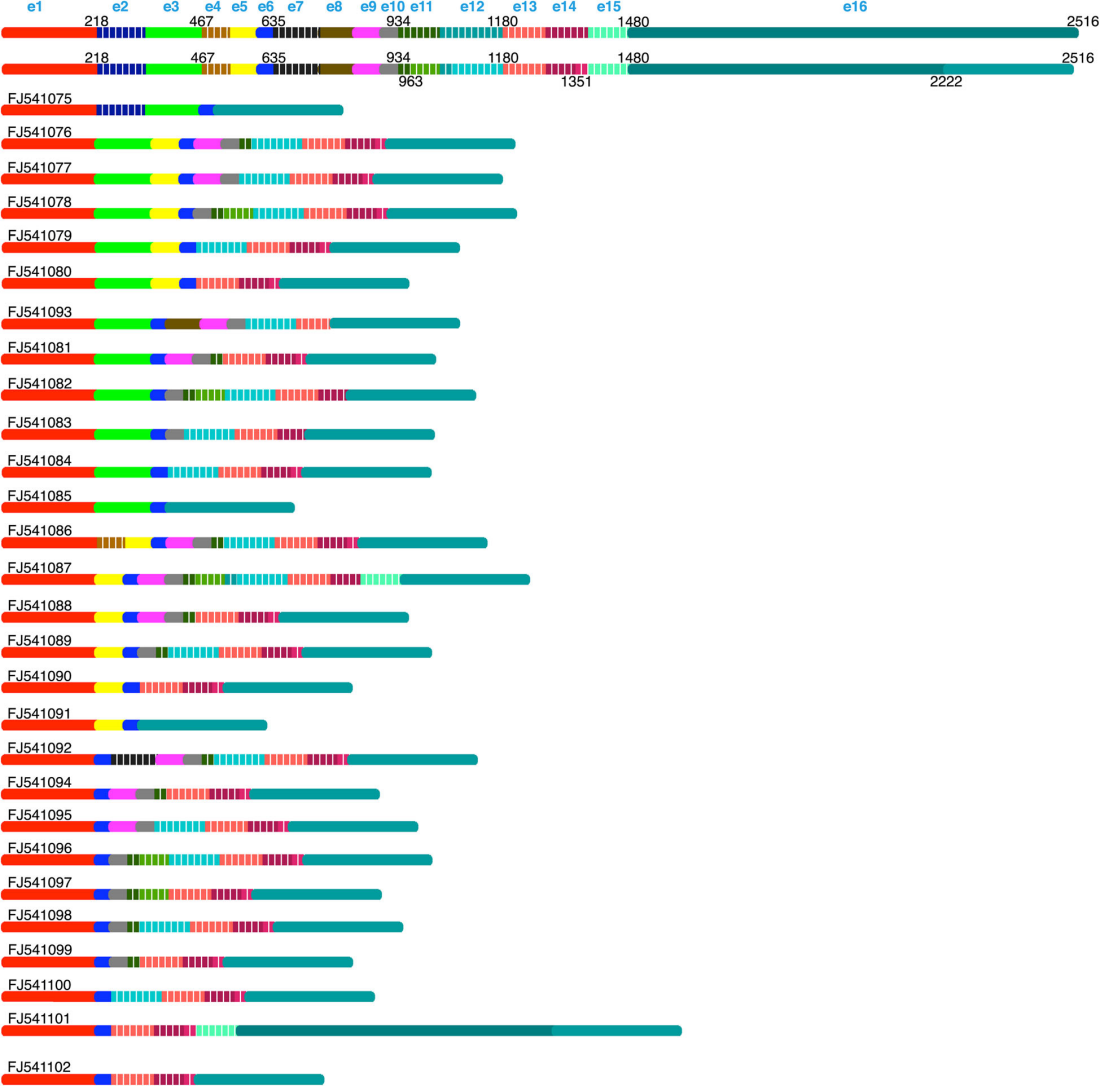
Representation of 28 *Pirmy-like* RNAs (FJ541075-FJ541102). Top two lines show the representation of all exons present together according to their order in the *Pirmy-*like RNAs as e1, e2, etc. Putative nucleotide positions in a scenario wherein all the exons are present are indicated. Different *Pirmy-*like RNAs have been arranged in linear order. Exons in dashed lines are specific to these 28 *Pirmy-*like RNAs, whereas other exons are common to *Pirmy* splice variants (Fig. 2) and the *Pirmy-*like RNAs

*Pirmy* was analysed further by RT-PCR. Two rounds of amplification using primers to the two ends of *Pirmy* yielded multiple products in testis and brain (Additional file 2: Fig. 2A). The PCR products from testis were cloned. Sanger sequencing of more than 1000 clones yielded 107 transcripts (NCBI accession numbers FJ541075-FJ541181), besides the one obtained by screening the testis cDNA library (Figs. 2B, C and 3). BLAST analysis of these transcripts against the NCBI genome database (GRCm39) showed that 79 transcripts (FJ541103-FJ541181) and *Pirmy* (DQ907162.1) were present at a single locus on mouse Y chromosome at 4341127-4381724 (Fig. 2B). These 79 transcripts could therefore be alternatively spliced isoforms of *Pirmy* (*Pirmy* splice variants)*.* The splice variants of *Pirmy* exhibited the full spectrum of splicing patterns like exon skipping, alternative 5′ and 3′ splice sites, mutually exclusive alternative exons, intron retention and combination of different splicing events (Additional file 3: Fig. S2). All the *Pirmy* splice variants contained consensus splice signal sequences (AG/GT) at all the intron-exon junctions (Additional file 4: Supplemental data sheet).

The remaining 28 of the 108 transcripts (FJ541075-FJ541102; Fig. 3) localize to different regions on mouse Y chromosome in 1–29 copies; these have been designated as *Pirmy*-like RNAs. Thus, even though *Pirmy* splice variants are found only at a single locus on Y, each exon from *Pirmy* is present in multiple copies on the Y chromosome. The 28 *Pirmy-*like RNAs contain different combinations of these exons at different loci in multiple copies on mouse Y.

### Expression of M34 in XY^RIII^qdel mice

Metaphase spreads from the XY^RIII^qdel mice showed a reduction in copy number of M34 on the Y chromosome (Additional file 5: Fig. S3A). Therefore, expression of M34 was then checked in testis and sperms of XY^RIII^ and XY^RIII^qdel mice by FISH. Dramatic reduction in fluorescence intensity was observed in testis and sperm of XY^RIII^qdel mice. However, sperms from epididymis of both XY^RIII^ and XY^RIII^qdel mice showed faint fluorescence intensity (Additional file 5: Fig. S3A). The subclones of M34 (Additional file 5: Fig. S3B) when used as probes on testis sections showed reduction in fluorescence intensity in XY^RIII^qdel mice (Additional file 5: Fig. S3C). We also checked the copy number of *Pirmy* in genomic DNA isolated from the XY^RIII^ and XY^RIII^qdel mice by real-time PCR using primers to exon 7; a significant reduction in copy number (*P* < 0.01) was observed in the XY^RIII^qdel genome (Additional file 6: Fig. S4).

### Many proteins coded by autosomal genes are deregulated in XY^RIII^qdel sperm proteome

Morphology- and motility-related abnormalities have been described in two strains of Y-deleted mice, the XY^RIII^qdel and B10.BR-Ydel [2, 30, 31]. We analysed the motility profile of sperms from XY^RIII^ and XY^RIII^qdel mice and observed a stark difference in motility pattern (Additional file 8, 9: Movies S1, S2 respectively). Spermatozoa from XY^RIII^ mice show linear progressive motion whereas sperms from XY^RIII^qdel mice show rapid flagellar movement with non-linear and non-progressive motion. Most of the spermatozoa from XY^RIII^qdel mice stall at the same position with no linear displacement. In order to understand the connection between the Y-deletion and sperm abnormalities, we performed comparative sperm proteome analysis between normal mice (XY^RIII^) and the XY^RIII^qdel mice by 2D-PAGE and mass spectrometry using protocols standardized in the laboratory [32].

**Fig. 4:**
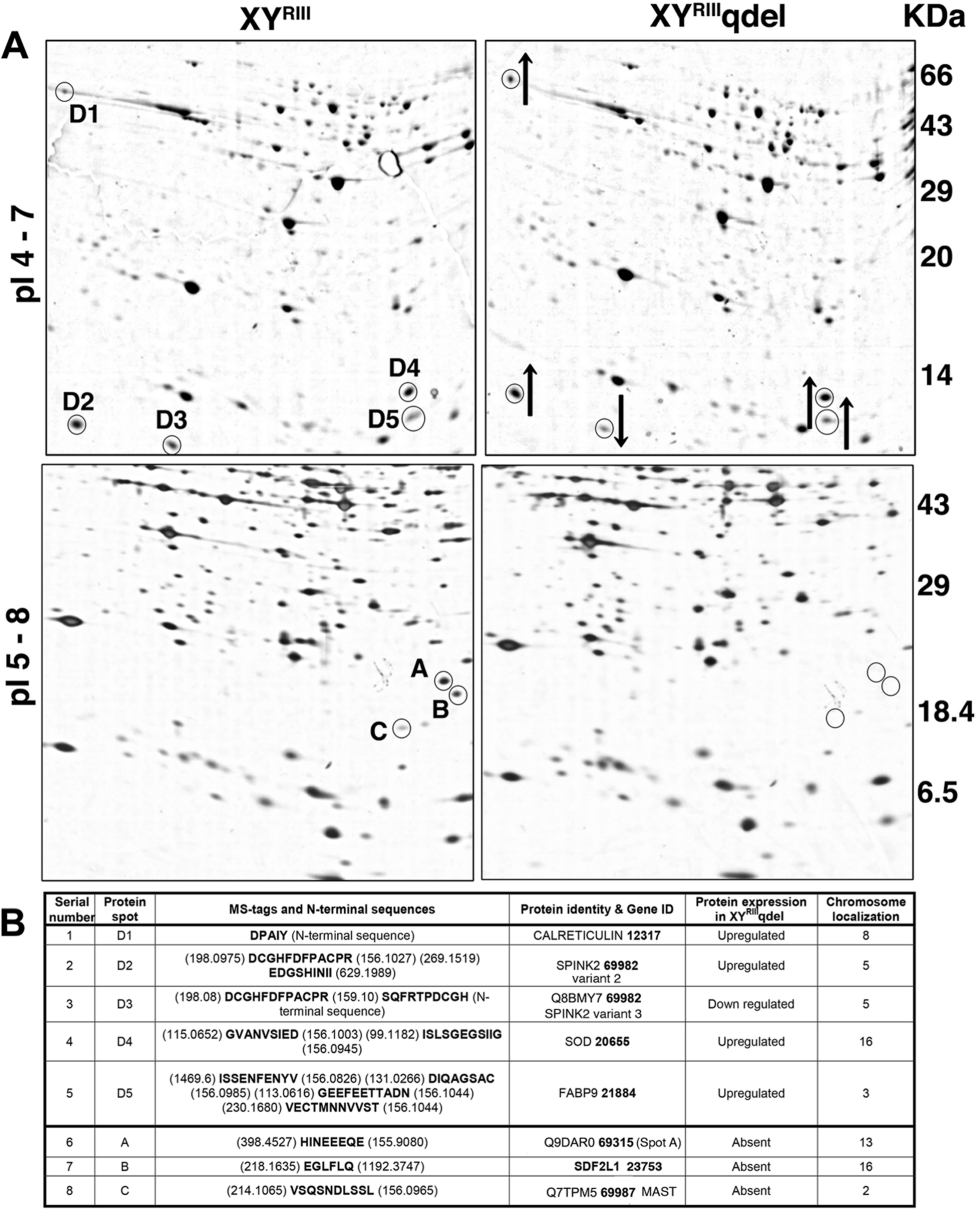
Sperm proteins are deregulated in XY^RIII^qdel mice. **A** Sperm lysates from the wild type XY^RIII^ strain and the mutant XY^RIII^qdel mice were separated by 2D-PAGE in the pI ranges of 4–7 and 5–8 on 8–20% gradient gels (see also Additional file 10: Fig. S5A). Five differentially expressed proteins, D1 to D5 - Calreticulin, SPINK2 variant 2, SPINK2 variant 3, SOD and FABP9 respectively, were identified by mass spectrometry analysis in the 4–7 pI range, of which 4 were upregulated (upward arrow) and one downregulated (downward arrow). Three proteins, A, B and C—spot A, SDF2L1 and MAST were not detectable in the 5–8 pI range in XY^RIII^qdel compared to the XY^RIII^ sperm lysate. **B** List of the differentially expressed proteins identified in the proteomics screen is given in the table along with MS tags and N-terminal sequences. Genes corresponding to all the differentially expressed proteins in XY^RIII^qdel localized to different autosomes

This analysis identified five protein spots that were differentially expressed in the pI range of 4–7 (D1–D5) and three proteins in the pI range of 5–8 (A, B, C) (Fig. 4A). Surprisingly four of these, i.e. calreticulin (D1), Serine Peptidase Inhibitor Kazal type II (SPINK2)/Acrosin-Trypsin inhibitor variant 2 (D2), Cu/Zn superoxide dismutase [SOD (D4)] and fatty acid-binding protein 9 [FABP9 (D5)], were upregulated in XY^RIII^qdel sperms compared to XY^RIII^ sperms (Fig. 4A, Additional file 10: Fig. S5A). A novel shorter isoform of SPINK2, i.e. SPINK2 variant3 [Q8BMY(D3)] which was shorter by 27 amino acids at the N-terminal end, was downregulated in XY^RIII^qdel sperms (Fig. 4A, Additional file 10: Fig. S5A). Three proteins were not detectable in XY^RIII^qdel sperms in the pI range of 5–8 (Fig. 4A). Two of these were reported as hypothetical proteins in the NCBI database (A -1700001 L19 Riken cDNA and C -1700026 L06 Riken cDNA). We have identified the proteins corresponding to the above Riken cDNAs. These have been deposited in the Uniprot database with accession numbers Q9DAR0 (A) and Q7TPM5 (C/MAST [32]) respectively. The third protein was identified as stromal cell-derived factor 2 like 1 [SDF2L1 (B)]. Expression of four of the eight differentially expressed proteins was also confirmed by western blotting in testis and sperms (Additional file 10: Fig. S5B, [32]). Surprisingly, all the eight genes corresponding to the differentially expressed sperm proteins localized to different autosomes (Fig. 4B).

Next, we analysed the expression of the transcripts corresponding to the protein spots in testis by real-time PCR/northern blot analysis (Additional file 10: Fig. S5C, S5D). Although the proteins Q9DAR0 (A), SDF2L1 (B) and MAST (C) were not detectable in sperms of XY^RIII^qdel, the corresponding RNAs were present in testis. The transcripts of SDF2L1, MAST, calreticulin and SPINK2 variant 2 proteins were upregulated in XY^RIII^qdel testis. In contrast, transcripts of Q9DAR0 and Spink2 variant 3 did not show significant quantitative difference between the two (Additional file 10: Fig. S5C). Northern blot analysis of *Sod* and *Fabp9* showed upregulated expression in XY^RIII^qdel testis (Additional file 10: Fig. S5D).

### UTRs of deregulated autosomal genes show homology to 108 transcripts

**Fig. 5:**
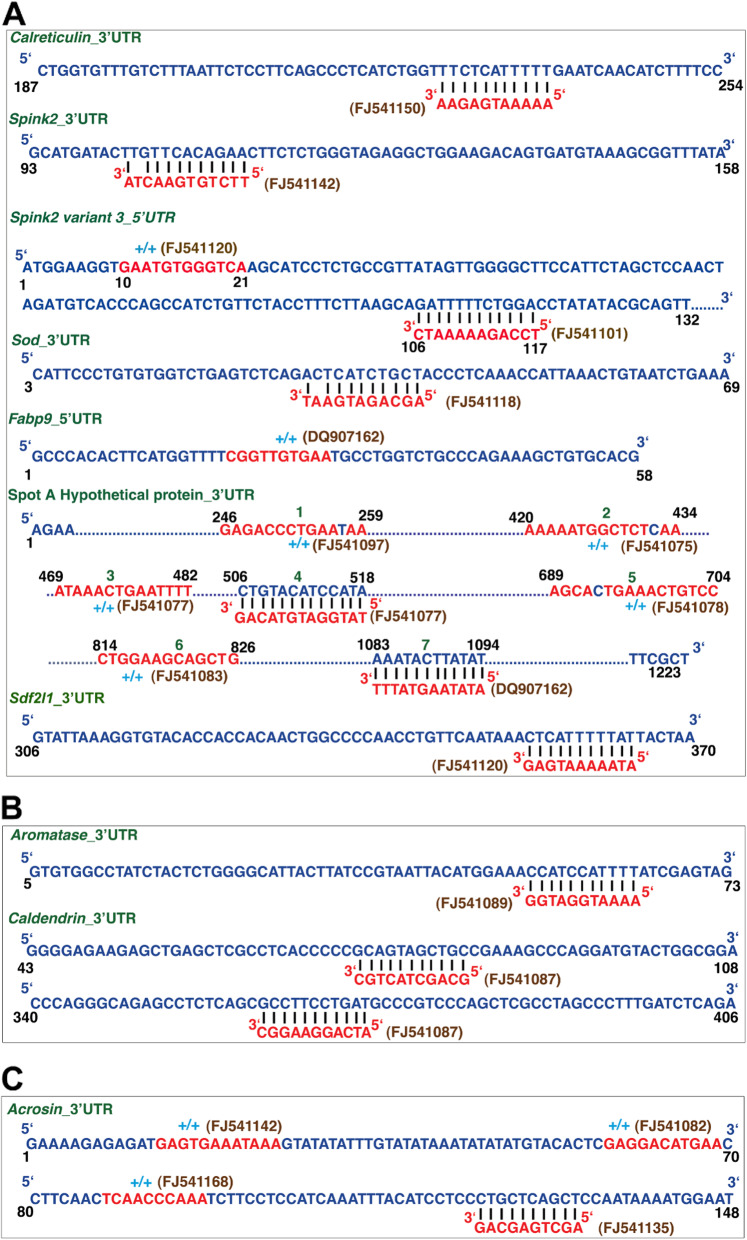
Localization of *Pirmy* transcripts to UTRs of deregulated genes. Panel **A** shows the UTR regions of the deregulated genes identified in the proteomics screen with the sequences homologous to *Pirmy* and *Pirmy-*like RNAs highlighted in red. Both +/+ and +/− homologies are observed. The splice isoforms of *Pirmy* and *Pirmy-*like RNAs are indicated in brown and the gene names in green colour. Seven homologous stretches were found in the 3′UTR of the spot A hypothetical protein. **B** Two deregulated genes (aromatase and caldendrin) were identified independent of the proteomics screen, which also harbour homology to *Pirmy-*like RNAs. **C** Acrosin identified from literature survey also harbours homologies to *Pirmy* and *Pirmy-*like RNAs. Acrosin harboured four homologous stretches in its 3′UTR

The fact that a few autosomal genes were deregulated when there was a deletion on the Y chromosome prompted us to investigate the mechanism behind this puzzling observation. We hypothesized that *Pirmy* and *Pirmy*-like RNAs that show reduction in copy number in the genome (Additional file 6: Fig. S4) and reduction in transcription in XY^RIII^qdel, could be regulating autosomal gene expression in testis. BLAST analysis of the 108 transcripts against the 3′ and 5′ UTRs of the deregulated genes revealed short stretches of homology ranging in size from 10 to 16 nucleotides (0-1 mismatch), in either +/+ or +/− orientations. This analysis identified 14 different hits in the UTRs of the deregulated genes. Of these, 7 were from *Pirmy* splice variants and 7 from the *Pirmy*-like RNAs respectively (Fig. 5). There are as many as 7 hits from *Pirmy* and *Pirmy*-like RNAs in the 3′ UTR of Q9DAR0 (hypothetical protein Spot A) (Fig. 5A). Aromatase was reported to be deregulated in B10.BR-Ydel mice [33]. We found upregulated expression of aromatase in XY^RIII^qdel mice testis compared to XY^RIII^ (data not shown). Earlier study in the lab reported upregulation of caldendrin in XY^RIII^qdel sperm compared to XY^RIII^ [32]. Acrosin is downregulated in B10.BR-Ydel sperm [13]. These three proteins which were reported to be deregulated in the two strains of Y-deleted mice also have homology to *Pirmy* and *Pirmy*-like RNAs in their UTRs (Fig. 5B, C). Homology between Y-derived transcripts and UTRs of deregulated autosomal genes indicates interactions between genes on the Y chromosome and autosomes in mouse testis. A BLAST against transcripts of the deregulated genes showed homology to the coding regions also; however, more stringent BLAST parameters of 10–16 nucleotide homology with > 95% identity showed homology to the UTRs alone.

**Fig. 6:**
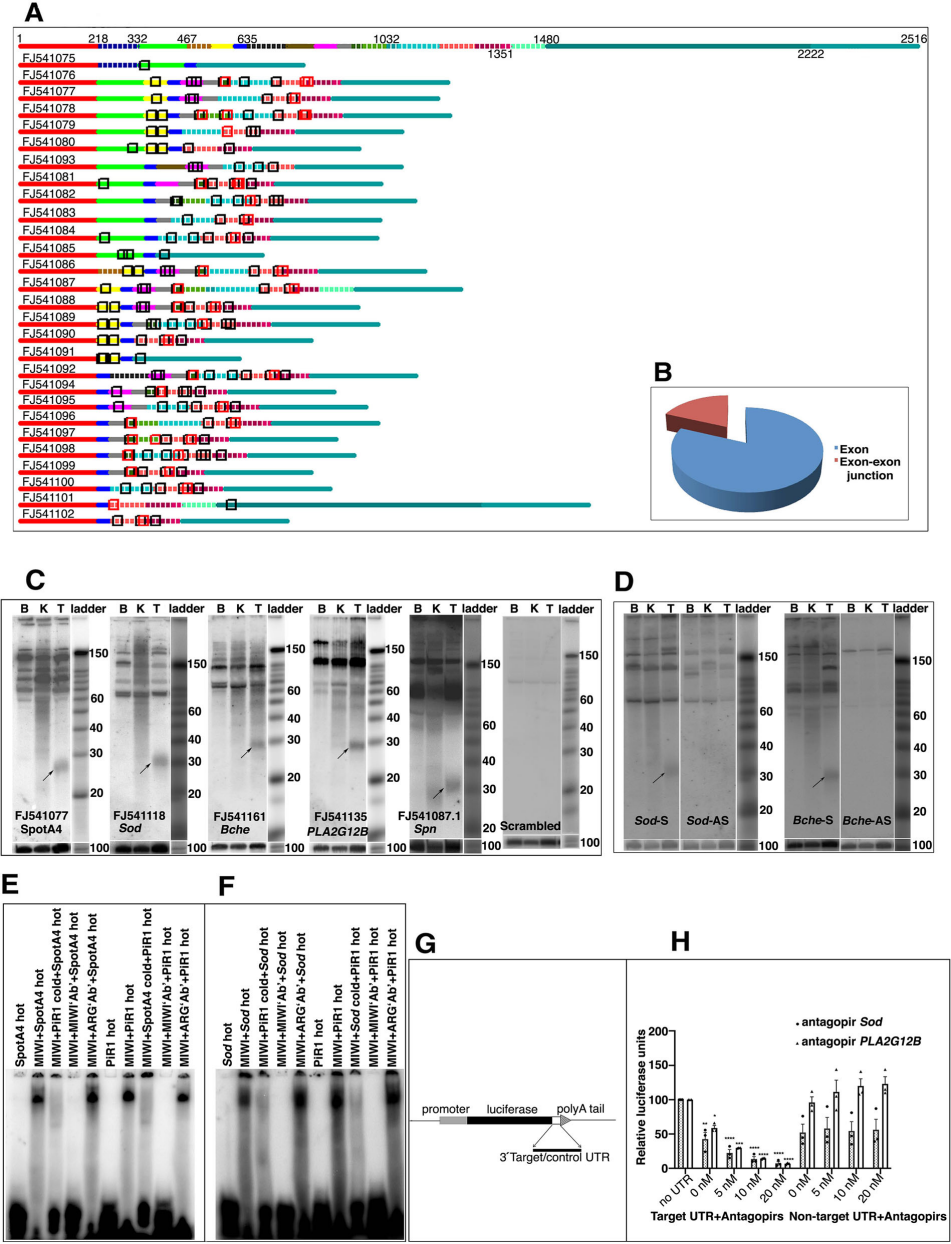
Identification of piRNAs in *Pirmy* and *Pirmy-*like transcripts. **A** Short stretches of homology identified in the UTRs are represented on the set of 28 *Pirmy-*like RNAs. The boxes highlighted in black match within exons and boxes highlighted in red match the exon-exon junctions. Line 1 indicates the nucleotide positions as in Fig. 3. **B** Pie chart shows ~ 81% of the matches within exons and ~ 19% at exon-exon junctions. **C** Use of sequences homologous to 3′ UTRs of a hypothetical protein (SpotA4), *Sod*, *Bche*, *PLA2G12B* and *Spn* as probes (Additional file 13: Fig. S6) on small RNA northern blots (3 experiments) shows testis-specific signals of ~ 30 nt size (indicated by arrows), which correspond to the size of piRNAs. Control blot using a scrambled oligonucleotide probe shows no signal at ~ 30 nt size. **D** Hybridization using probes from sense (S) and antisense (AS) strands of *Sod* and *Bche* (Additional file 13: Fig. S6) shows differential transcription from the two strands under identical conditions. Lower panels in **C**, **D** show loading control using U6 probe (B—brain, K—kidney, T—testis, Ladder—decade marker). **E, F** EMSA using RNA oligonucleotide sequences from FJ541077 (**E**) and FJ541118 (**F**) that have homology to UTRs of the genes of hypothetical protein spot A (A4) and *Sod* respectively*.* These oligonucleotides and piR1, a known piRNA, showed the shift in mobility with recombinant MIWI protein. Gel shifts obtained with RNA oligonucleotides from spotA4 and *Sod* were competed out by cold piR1 and vice versa. Pre-incubation with MIWI antibody abolished the gel shift whereas pre-incubation with argonaute 3 antibody (ARG) did not, indicating specificity of binding. The small RNA northern blots, EMSA experiments were repeated three times each. **G** Schematic representation of 3′UTR reporter constructs. **H** The concentration-dependent reduction in luciferase expression when target UTRs of *Sod* and *PLA2G12B* are treated with antagopirs (0–20 nM). A non-target UTR (*Cdc2l1*) did not show a difference (*n* = 3; *** *P* < 0.001; **P < 0.01; *P < 0.05)

Furthermore, we performed BLAST analysis of all the *Pirmy* and *Pirmy*-like RNAs against the entire UTR database. This identified small stretches of homology in the UTRs of a number of genes across different species. The homologous sequences (16–30 nt) localized to both exons and exon-exon junctions of *Pirmy* and *Pirmy*-like RNAs, with one or two mismatches. Representation of UTR homologies to these ncRNAs are shown in Fig. 6A. We identified 372 unique homologous stretches in *Pirmy* and *Pirmy*-like RNAs. Of these 302 (81.19%) localized to the exons and 70 (18.82%) to the exon-exon junctions (Fig. 6B).

### Identification of ~ 30 nt RNAs from *Pirmy* and *Pirmy*-like RNAs

To check if these short stretches of homologies correspond to small RNAs, few representative oligonucleotide sequences from the ncRNAs with homology to different UTRs were used as probes (Additional file 13: Fig. S6) on small RNA northern blots. Two genes were chosen from the deregulated proteins Q9DAR0 (Spot A) and superoxide dismutase (SOD); three genes (butyrylcholinesterase (*Bche*), phospholipase A2 group XIIB *(PLA2G12B)* and sialophorin *(Spn)*) were chosen from the BLAST output against entire UTR database. All the above probes elicited approximately 26–30-nt-long testis-specific signals, of the size of piRNAs (Fig. 6C). Inclusion of small RNA from Y-del testis in the northern blots did not show any appreciable change in the intensity of the ~ 30 nt signals when homologous sequences from the UTRs of Prot A1, Prot A4, Prot A3, *Sod*, *Bche*, *Mads* and *Oosp1* were used as probes (Additional file 14: Fig. S7).

To confirm that these homologous sequences are indeed piRNAs, different experiments were designed. As the antiparallel strands of DNA are reported to express different levels of piRNA [34], differential expression from the antiparallel strands were studied using sense (S) and antisense (AS) probes designed to homologous stretches in the 3′ UTRs of *Sod* and *Bche* (Additional file 13: Fig. S6). Identical experimental conditions showed differential expression of these ~ 30 nt species of RNAs from the two strands of DNA (Fig. 6D), further indicating that these short RNAs could be piRNAs.

As piRNAs are PIWI/MIWI-binding small RNAs, electrophoretic mobility shift assay (EMSA) using the *Pirmy*-derived oligonucleotides and recombinant MIWI protein was done to check if the sequences with homologies to UTRs of different genes are indeed piRNAs. Representative gel shifts using oligonucleotides from UTRs of Q9DAR0 (SpotA4) and *Sod* are depicted in Fig. 6E and F respectively. piR1 [34], a known piRNA, served as the positive control. Specificity of binding was indicated by the use of corresponding cold competitors as described in the legend to Fig. 6E and F. The piRNA-derived oligonucleotides competed out binding of piR1 to MIWI protein and vice versa. This confirmed that these oligonucleotides are indeed MIWI-binding RNAs and therefore piRNAs. The mobility shift was also competed out by MIWI antibody while Argonaute 3 antibody did not alter the mobility of the gel-shifted band obtained with MIWI indicating specificity of binding (Fig. 6E, F). These experiments provide further evidence that these short RNA sequences are piRNAs.

### *Pirmy* and *Pirmy*-like RNAs identify piRNAs from NCBI Sequence Read Archive (SRA) database

Next line of evidence to the fact that these short stretches of homologies are piRNAs came from the NCBI SRA database for piRNAs. BLAST analysis using *Pirmy* and *Pirmy*-like RNAs (the 108 transcripts) identified a total of 93 piRNAs in the piRNA-SRA databases SRP001701 and SRP000623 with 100% identity, with the length of match ranging from 22 to 35 (Additional file 16: Table S1). When we BLASTed the aligned regions of these 93 piRNAs against the mouse genome database, 79 of them mapped only to the Y chromosome with 100% identity and coverage. These are found in 1–246 copies on the mouse Y chromosome. This analysis further confirmed derivation of piRNAs from mouse Y chromosome.

### Antagopirs downregulate reporter gene expression

Complementary oligonucleotides synthesized to piRNA sequences present in UTRs of *Sod* and *PLA2G12B* were designated as antagopirs (Additional file 13: Fig. S6). These UTRs were cloned 3′ to the Luciferase reporter constructs (Fig. 6G). Treatment with increasing concentrations of antagopirs, i.e. 5 nM, 10 nM and 20 nM caused concentration-dependent reduction in Luciferase expression (Fig. 6H). The antagopirs to *Sod* and *PLA2G12B* did not have an effect when the UTR from a non-target gene (*Cdc2l1)* was used. Thus, we demonstrate that antagopirs to piRNAs modulate gene expression in vitro.

This study therefore, paved the way for a series of novel and exciting observations. We have identified a novel, polyadenylated lncRNA (*Pirmy*) expressed from mouse Yq in testis that shows a large number of splice variants. The Y-derived *Pirmy* and *Pirmy*-like RNAs harbour piRNAs that have homology to the UTRs of few autosomal genes expressed in mouse testis. The proteins expressed from these autosomal genes are deregulated in sperms of Yq-deleted mice and appear to be controlled by piRNAs generated from MSYq-derived lncRNAs. Consolidation of the results from the proteomics analysis and the molecular studies suggested that piRNAs generated from male-specific mouse MSYq regulate expression of multiple autosomal genes in testis. Partial deletion of MSYq resulted in deregulation of these proteins leading to sperm anomalies and subfertility. Thus, subfertility in mice appears to be a polygenic phenomenon that is regulated epistatically by the Y chromosome.

## Discussion

Y chromosomes harbour genes for male determination and male fertility. Yet the role of Y chromosomal repeats in male fertility and spermatogenesis remains quite enigmatic. In this study, we elucidate the role in male fertility of a species-specific repeat, M34, from mouse Y long arm, which is transcribed in mouse testis. Transcription from repeats on mouse Y chromosome has been reported earlier. Testis-specific transcription of a family of poly(A)RNAs from the mouse Y chromosome was first reported by Bishop and Hatat using a multicopy Y-derived probe, pY353/B [2, 35]. Subsequently, more transcripts were identified from repeat sequences localizing to the Y chromosome, in mouse testis [9, 10, 19, 36]. The report of *Pirmy* and *Pirmy*-like RNAs described in this study adds to the repertoire of transcribed repeats on mouse Y.

### Novel noncoding RNAs from mouse Y long arm

The 108 *Pirmy* and *Pirmy*-like RNAs in the present study were discovered serendipitously by cloning and sequencing of the multiple RT-PCR products obtained using primers to the initial and terminal exons of *Pirmy* (DQ907162). As the primers were restricted to just two of the exons, it is possible that we might discover more splice variants and more *Pirmy*-like RNAs using primers to different combinations of exons for RT-PCR amplification. Alternative splicing has been reported in ncRNAs [37]; yet, such extensive splicing as observed in our study has not been reported for any of them. Very few polymorphically spliced genes have been described earlier from sex chromosomes, particularly the Y [19, 21, 36, 38]. Thus, the 80 splice variants of *Pirmy* in this study appear to be by far the maximum number of isoforms characterized from a single ncRNA by alternative splicing. The consensus splice signal sequences present at the intron-exon junctions in all *Pirmy* splice variants reaffirm programmed splicing events. It is possible that *Pirmy* locus is similar to *Orly*, to the extent that it contains a partial copy of *Sly* along with other sequences. *Orly* is a chimeric transcript that contains exons from different genes like *Ssty1*, *Asty* and *Sly* [36].

### The deregulated proteins contribute to sperm phenotype in XY^RIII^qdel mice

We speculate that the deregulated proteins identified in the current study are at least partially responsible for the sperm morphology, motility and sex-ratio phenotypes observed in XY^RIII^qdel mice. For example, FABP9 that localizes to the perforatorium, the subacrosomal region in spermatozoa with falciform head shapes [39–41], is upregulated in XY^RIII^qdel sperms and therefore could be a factor contributing to misshapen heads. Kherraf and colleagues [42] observed grossly misshapen sperm heads and reduced motility in Spink2 variant 2 knockout mice. The deregulated expression of Spink2 isoforms also could therefore contribute to sperm head morphological abnormalities and reduced sperm motility.

Calreticulin is a calcium store-associated protein with sperm functions such as hyperactivated motility, capacitation and acrosome reaction [43]. The overexpression of it in XY^RIII^qdel spermatozoa could affect motility and fertilization. SOD, which is associated with sperm count and overall motility [44], is upregulated in XY^RIII^qdel. MAST is a novel protein that is not detectable in the XY^RIII^qdel sperm proteome, but localizes to both the acrosome and sperm tail, indicating a role in sperm motility and penetration of the egg [32]. Bioinformatic analysis of the novel protein Q9DAR0 (spot A) predicts it as a cilia-related gene [45]. Based on this information putative role of this protein in sperm motility can be envisaged. The ER stress-inducible SDF2L1 [46] is not detectable in XY^RIII^qdel sperms. Hence, the XY^RIII^qdel spermatozoa which lack the stress response protein may be more susceptible to stress induced damages. The subsequent cascade of events could result in subfertility. Thus, looking into the functions of the proteins identified as deregulated in XY^RIII^qdel sperms, it appears that they play a role in sperm morphology, motility and other sperm functions. The localization of majority of the morphology, motility-related proteins identified in this study, to sperm head and/or tail is as shown in Additional file 17: Figure S8.

Aromatase which is overexpressed in XY^RIII^qdel (our unpublished observation), and B10.BR- [47] catalyses the irreversible conversion of androgens to estrogens [48]. However, the effect of increased aromatase on skewed sex ratio in the XY^RIII^qdel mice is not clear. The differential motility of X- and Y-bearing sperms described by Ellis and colleagues could explain the skewed sex ratio noted in the XY^RIII^qdel mice [31]. Two other acrosomal proteins are reported to be deregulated in Yq-deleted mice. B10.BR Y-del mice show a reduction in expression of acrosin [41]. Spermatozoa lacking acrosin (acrosin^+/−^) exhibit delayed fertilization [42–44]. Caldendrin that is upregulated in XY^RIII^qdel sperm [32] is yet another protein that localizes to acrosome in rats and is considered to be a stimulus-dependent regulator of calcium [49].

### Connection between autosomal genes and the Y chromosome

The identification of deregulated proteins, for which the corresponding genes localize to autosomes, in a Y-deletion mutant was a surprise. Presence of small stretches of homology in the UTRs of these transcripts from the *Pirmy* and *Pirmy*-like RNAs established the connection between the two. Small RNAs of the size of 30 nucleotides identified on using these homologous stretches as probes hinted that these could be piRNAs. Small RNAs of the size of ~ 27–32 nucleotides that bind PIWI protein are classified as piRNAs [34]. Association with PIWI, but not AGO proteins is a characteristic feature of piRNAs [50]. The fact that MIWI antibody and not the argonaute antibody prevented binding of the putative piRNAs strengthens the argument that these are indeed piRNAs. *Pirmy* and *Pirmy*-like RNAs also identified piRNAs in SRA databases which mapped exclusively to mouse Y chromosome. Differential expression from the two strands of DNA is another characteristic feature of piRNAs [34] that is observed in the representative piRNAs reported here in the current study (Fig. 6D). The presence of piRNAs in the UTRs of genes corresponding to deregulated proteins suggests putative regulation of these autosomal genes by Y chromosome-derived piRNAs. Y chromosome-derived piRNAs have been described from multicopy gene families localizing to mouse Y chromosome [51]. piRNA-dependent regulation of mRNAs and lncRNAs has been reported by Watanabe et al. [52]. The concentration-dependent reduction of Luciferase expression by antagopirs corroborates the regulation of these genes by Yq-derived piRNAs.

Proteins from three other autosomal genes, caldendrin, acrosin and aromatase, are also deregulated in Yq-deleted mice besides the ones identified in the proteomics screen. Therefore, it is not surprising to find sequences homologous to *Pirmy* and *Pirmy*-like RNAs in the UTRs of these three genes, suggesting Y-mediated regulation for these genes as well. Ellis and colleagues also observed up- or downregulation of genes from the X-chromosome and autosomes in testes of mice with deletions of Y long arm using a microarray approach [53]. Homology between UTRs of some of the above genes and the *Pirmy* and *Pirmy*-like RNAs (Additional file 18: Table S2) further strengthens the hypothesis of putative regulation of genes located elsewhere in the genome by Y chromosomal repeats. Ten of the eleven genes identified in our study and 6/10 genes identified in the microarray analysis by Ellis et al. [53] have homologies to the *Pirmy* and *Pirmy*-like RNAs in the UTRs of testis-expressed isoforms of these genes. The regulation by *Pirmy* and *Pirmy*-like RNAs therefore shows a bias towards genes expressed in testis, although several of the genes show ubiquitous expression including testis.

Interestingly, northern blot analysis including small RNA from XY^RIII^qdel testis did not show a visible reduction in piRNA signals when compared to XY^RIII^ testis, on use of at least 7 different UTR homologous sequences as probes. This could be because the differences significant at the physiological levels may not be identified with techniques such as northern blotting. This could be possibly due to the effect of piRNAs on chromatin structure. Watanabe and colleagues have shown that piRNAs mediate degradation of a large number of mRNAs and lncRNAs in mouse late spermatocytes [52]. Further, they go on to show that a quarter of lncRNAs are upregulated in mice deficient in the piRNA pathway [52]. Studies by Cocquet et al. [17] and Ellis et al. [53] show that MSYq deletion (XY^RIII^qdel) shows upregulation of several X- and Y chromosomal genes. If some of the piRNAs are derived from the genes that are upregulated, such ones will not show a reduction of the 28-30 nt signals on northern blots.

Functions of Y chromosome have been elucidated using different deletions of the chromosome in the past. Naturally occurring deletions in the euchromatic long arm of Y chromosome in azoospermic men showed the involvement of this region in human male infertility [54]. *Drosophila melanogaster* males with deletions of different regions of the Y chromosome show absence of several sperm axoneme proteins [55]. Previous studies in the lab elucidated an example of an intronless Yq-derived ncRNA-mediated regulation of an autosomal gene, *CDC2L2*, via trans-splicing in human testis [27]. Mice with partial or total deletions of Y long arm show deregulation of testicular gene expression and subfertility/sterility [17, 53]. The noncoding RNAs described in this study, *Pirmy* and *Pirmy*-like RNAs, appear to modulate the expression of the deregulated proteins in Yq-deletion mutant mouse. Knockout of all the *Pirmy* exons would have unequivocally established the role of *Pirmy* and *Pirmy*-like RNAs in male fertility in mouse. But knocking out of all these transcripts is practically not possible, due to the presence of multiple copies in different combinations of these exons and introns. Even though *Pirmy* and its splice variants are present at a single locus on the Y chromosome, the exons contained within these transcripts are present on the Y chromosome as *Pirmy*-like RNAs in multiple copies at different loci. Therefore, knocking out the *Pirmy* locus alone will not have an all or none effect. The use of mice with larger Yq deletions would further establish the link between Y chromosome and these piRNAs. The role of mouse Yq repeats in the current study therefore reveals a novel pathway for the regulation of autosomal genes by Y chromosome, mediated by piRNAs, in male reproduction. Therefore, consolidation of the observations in the lab shows that Y chromosome regulates autosomal genes expressed in testis using distinct mechanisms viz., trans-splicing [27] and piRNA-mediated regulation in the current study.

### Sperm-related phenotypes in Yq-deleted mice resemble those described in cross-species male-sterile hybrids

Comparative sperm proteomics analysis in our study portrays involvement of multiple autosomal genes in subfertility. The regulation of autosomal gene expression appears to be relaxed in sperms of Yq-deleted mice [23, 53]. This reflects a connection between the Y chromosome and autosomes. In fact, as suggested by Piergentili, Y chromosome could be a major modulator of gene expression [5]. Our results seem to provide explanation for some of the earlier classical observations of mice with different Y chromosomal deletions exhibiting subfertility/sterility along with sperm morphological abnormalities, fewer motile sperms, sex ratio skewed towards females, etc. Similar phenotypes are also observed in cross-species male-sterile hybrids of *Drosophila* and mouse [5, 56–62]. Y chromosome has also been implicated in the male sterility phenotype of these interspecies hybrids [59, 61, 63–65]. Thus, the phenotypes observed in cross-species male-sterile hybrids and the Y-deletion mutants are comparable. Introduction of Y chromosomes into different genetic backgrounds of *Drosophila* resulted in deregulated expression of hundreds of genes localizing to the X-chromosome and autosomes [66, 67]. It has also been proposed that incompatibility between the Y chromosomes and different autosomes could result in the hybrid dysgenesis of sperm-related phenotypes observed in *Drosophila* [64]. Zouros and colleagues also suggested the presence of epistatic networks in interspecies hybrids, based on the fact that homospecific combination of alleles at a given set of loci could sustain normal development, but heterospecific combinations could not [63, 68, 69]. This early hypothesis seems to be amply supported by our study. Further, our results elucidate the Y-derived piRNAs as the genetic basis of epistatic interactions between Y chromosome and autosomes in mouse. Our results also suggest for the first time, the mechanism of piRNA-mediated regulation of autosomal genes involved in spermiogenesis and male fertility. This, to our knowledge is the first report on possible regulation of autosomal genes involved in male fertility and spermiogenesis, mediated by Y-encoded small RNAs/piRNAs in any species.

## Conclusions

In brief, the XY^RIII^qdel mutant strain of mouse, where there is a partial deletion of long arm of the Y chromosome, exhibit sperm morphological and motility-related aberrations and subfertility [2]. A comparative sperm proteomic profiling of the XY^RIII^ and XY^RIII^qdel mice captured few differentially expressed proteins that could partially account for the aberrant sperm phenotype. Surprisingly, genes corresponding to the deregulated proteins localized to autosomes and not to the deleted region of the Y chromosome. A search for the Y-autosome connections in mouse led to the identification of novel ncRNAs from mouse Y long arm that subsequently was shown to regulate the genes expressed in testis via piRNAs. Thus, adopting a top-down approach, we have established a novel mode of regulation of autosomal genes expressed in mouse testis by the Y chromosome and the biology behind the aberrant sperm phenotype in Yq-deleted mice.

Finally, evolutionary impact of novel genetic interactions or regulatory mechanisms such as those reported in this study could be significant. The generation of piRNAs from species-specific repeats on mouse Y chromosome that apparently regulate autosomal gene expression in testis raises more questions in the field of speciation and evolution. Do mutations in the Y chromosomal repeats collapse the poise of the species? Are species-specific repeats on the Y chromosome the fulcrum on which rests the fine balance between species identity and evolution?

## Methods

### Animals and reagents

The XY^RIII^ strain (wild type) and the XY^RIII^qdel strain (Y-deletion mutant) of mice in the outbred MF1 background used in the study was a gift from Prof. Paul S Burgoyne, MRC, UK. The animals were maintained in the animal house facility of our institute (CCMB) for our experiments. The recombinant construct in pAAV-IRES-hrGFP from which MIWI protein was isolated was a gift from Dr. Arvind Kumar, CCMB, Hyderabad. The GC-1spg cell line (ATCC CRL-2053) was obtained as a gift from Prof. MRS Rao, JNCASR, Bangalore, India. Reagents used in the study and their catalogue number are mentioned in the respective experiments described in the “Methods” section.

### Fluorescence in situ hybridization

Testes collected from different developmental stages (18.5 days of embryonic stage, new born, 1 month and 2 months (adult)) of XY^RIII^ strain were processed for tissue FISH with the genomic clone M34 (DQ907163), following published protocols [27]. Chromosomal localization using FISH was done with the genomic clone DQ907163 and the cDNA clone DQ907162 on metaphase spreads prepared from bone marrow of XY^RIII^ and XY^RIII^qdel strains*.* Briefly, mouse bone marrow was flushed out into tissue culture medium DMEM (Dulbecco) and incubated at 37 °C for 2–4 h with colcemid (0.5μg/ml of culture) for the last 2 h to get a good frequency of metaphase spreads. Chromosomal FISH was done using protocols standardized in the laboratory earlier [27].

### Identification of cDNA using M34 (DQ907163)

An amplified mouse testis cDNA library (mouse testis MATCHMAKER cDNA Library – Clontech, Cat # ML 4015AH) was screened with the male-specific ES cell EST (CA533654) with sequence homology to the M34 genomic clone DQ907163 (Fig. 1E) as per manufacturer’s protocol. Mouse testis cDNA Library was screened at a stringency of 2 × SSC, 0.1% SDS at 65 °C for 10 min (washing conditions). A total of 2 × 10^5^ colonies was screened to obtain 18 clones after tertiary screening. Male specificity of the positive clones from the library was determined using southern blots containing mouse male and female DNA. Sanger sequencing of all the 18 clones confirmed them to be the same. The sequence is named as *Pirmy* (piRNA from mouse Y chromosome) and submitted to the NCBI database with accession number DQ907162**.**

### Identification of splice variants

Total RNA (1 μg) isolated from brain and testes tissues of XY^RIII^ strain of mouse were reverse transcribed with the SuperscriptII Reverse Transcriptase enzyme (Thermo Fisher Scientific Cat # 18064022), using oligo (dT) primers and random hexamers. PCR with GAPDH primers was used for checking the quality and integrity of RNA. DQ907162 was amplified after two rounds of PCR using primers to the first and last exons. For the first round, forward (GTGTGACAGGGTGGGGAATC) and reverse primers (TTCCTGAAGATAGCACTTGTG), and the following conditions were used—initial denaturation 95 °C, 1 min, cycle denaturation 95 °C, 1 min, annealing 62 °C, 30 s and extension 72 °C for 2 min (35 cycles), final extension 72 °C for 7 min. The second round of amplification was done using nested primers GAGGACCGTATTCATGGAAGAG (forward) and GCAAATGGCTCACATCAGTGG (reverse) using initial denaturation at 95 °C for 1 min, cycle denaturation at 95 °C, 1 min, annealing at 66 °C for 30 s and extension at 72 °C for 2 min (38 cycles) and final extension at 72 °C for 7 min. The multiple products obtained after two rounds of PCR from both testis and brain (Fig. 2A) were cloned into pCR TOPO vector using TOPO TA cloning kit (Thermo Fisher Scientific Cat # 450641). Approximately 1000 clones were sequenced from testis, on a 3730 DNA Analyser (ABI Prism, Thermo Fisher) using sequencing kit BigDye Terminator V3.1 Cycle Sequencing kit (Thermo Fisher Cat # 4337457). These yielded 108 unique transcripts. BLAST analysis of these transcripts against the genomic sequences (GRCm39) at a stringency of > 97% localized 80 of these to a single locus on mouse Y chromosome (4341127-4381724) and the rest to multiple sites on Yq.

### Collection of sperm and CASA recording for assessing sperm motility

Adult male mice of approximately 3 months of age were sacrificed by cervical dislocation. Dissected cauda epididymides were washed in pre-warmed PBS and spermatozoa were collected by puncturing it with a needle. The sperms were allowed to ooze out of the cauda, in warm modified Krebs Ringer medium (NaCl—94.6 mM, KCl—4.78 mM, CaCl_2_—1.71 mM, KH_2_PO_4_—1.19 mM, MgSO_4_—1.19 mM, NaHCO_3_—25.07 mM, glucose—5.56 mM, sodium lactate—21.58 mM, sodium pyruvate—0.5 mM, HEPES Na^+^ salt—10 mM (pH 7.4), phenol red—0.001 gm and BSA (fraction V)—4 mg/ml). pH was adjusted to 7.4 after dissolving all the above components, except BSA. BSA was added at the end and the solution was placed in humidified CO_2_ incubator at 37 °C for at least 1 h. Comparable dilutions of the sperms from the XY^RIII^ and XY^RIII^qdel mice were dispersed into pre-warmed slide chambers and covered with cover slips. The cells were observed using a Computer Aided Sperm Analyzer (CASA) (Hamilton-Thorne, Maryland, USA) with settings specific for mouse sperm capture (HTM CEROS, version 12.0 L) and the captures were recorded through a CCD camera.

### Copy number estimation of *Pirmy* and *Pirmy*-like RNAs

Genomic DNA was isolated from testes of XY^RIII^ and XY^RIII^qdel mice (4 each) using phenol-chloroform method and quantified using a Nanodrop (NANODROP 2000, Thermo Fisher Scientific). Quantitative real-time PCR (LightCycler 480, Roche) was performed using SYBR green master mix (Roche Diagnostics Cat # 4707516001) with 2 ng of genomic DNA and a primer concentration of 200 nM per reaction. The primers used were as follows:

**Table:** 

*Pirmy* (exon 7)	Forward reverse	5′GTG CGG TTG TGA AGG TGT TC3′ 5′CCT CCA CCT TCC ATT CAC CC3′
*Gapdh*	Forward Reverse	5′ACG GGA AGC TCA CTG GCA TGG3′ 5′CAA CAG CGA CAC CCA CTC CTC3′

PCR conditions included an initial denaturation for 5 min at 95 °C followed by 45 cycles of denaturation at 95 °C for 10 s, annealing at 58 °C for 20 s and elongation at 72 °C for 30 s. The amplification of specific product was confirmed by melting curve profile (cooling the sample to 65 °C for 1 min and heating slowly with an increase in temperature of 5 °C at each step till 95 °C, with continuous measurement of fluorescence). The relative copy number of *Pirmy* and *Pirmy-*like RNAs was analysed based on Livak method (2^−ΔΔCt^).

### Sperm proteome analysis

Sperm were collected and processed for proteome analysis as per the protocol given in Bhattacharya et al. [32]. Briefly, sperm lysate (1 mg of cell weight per 5 μl of lysis buffer) was incubated on ice for 1 h to allow buffer to permeabilize and lyse the sample. Further, the sample was briefly sonicated on ice. The lysate was centrifuged for 15 min at 13,000 rpm at 4 °C. The supernatant was collected and was further taken for ultra-centrifugation at 55,000 rpm for 1 h at 4 °C. The clear lysate was collected in fresh tube and PMSF was added to a final concentration of 1 mM. The protein concentration in the cell lysate was estimated by bicinchoninic acid assay (Pierce, BCA protein assay kit Thermo Fisher Cat # 23225), following the manufacturer’s instructions in a micro titre plate. BSA was used as the standard for estimation. The proteins were separated in the first dimension on 4–7/5–8 IPG strips (Bio-Rad ReadyStrip IPG strips Cat # 1632001, 1632004). The strips were then loaded onto 8–20% gradient PAGE for separation on the second dimension. Protein spots were visualized by Coomassie Blue staining. Spot to spot matches were done to identify differences. Analysis of five sets of gels after normalization with control spots using PDQUEST software version 6.0 (Bio-Rad) identified the differential proteins. Measuring the optical density of these differentially expressed proteins in arbitrary units validated the quantitative differences (Additional file 10: Fig. S5A). These values were subjected to nonparametric Kruskal-Wallis H test and the levels of confidence determined by the Chi-squared test (75–95% degrees of confidence). Trypsin digested spots were processed to obtain the protein tags by MS analysis on a Hybrid Quadrupole TOF mass spectrometer (API QSTAR PULSAR i, PE SCIEX).

### Western blot analysis

Western blot analysis was performed as per the published protocol [32]. Antibodies used were SOD (Santacruz Cat # sc-17767), β-Tubulin (Santacruz Cat # sc-166729), Horse Raddish Peroxidase (HRP)-conjugated secondary antibody (Abcam Cat # ab97023) and FABP9 (R&D Systems Inc. Cat # - AF2750).

**Table:** 

Sr. No	Antibody	Primary antibody dilution	Secondary antibody dilution
1.	SOD	1:2000	1:3000
2.	FABP9	1:3000 along with 1/20 blocking agent	1:5000 along with 1/50 blocking agent

### Real-time PCR analysis of transcripts of differentially expressed proteins

The total RNA was extracted from adult mouse testes using Trizol (Invitrogen). One microgram of RNA was reverse transcribed to cDNA using Verso cDNA synthesis kit (Thermo Fisher Scientific Cat # AB1453A). The quality of each sample was checked by analysing housekeeping genes on agarose gel and qPCR melting curve analysis. cDNA samples from six mice that belong to the same group were then pooled together. qPCR was performed using SYBR green master mix (Roche Diagnostics Cat # 4707516001) and analysed in Roche Light Cycler LC480. The primer sequences corresponding to the transcripts are as follows:

**Table:** 

Riken cDNA 1700001 L19 (Q9DAR0) (A)	Forward reverse	5′CGA GGG CCA GAC AGG GAT TG3′ 5′CCC ATA GAC AGA GGA CAT CAG-3′
*Sdf2l1* (B)	Forward reverse	5′ACT TCC CGT CGC CGC TAT C-3′ 5′TGA CCG ACA GGA ACA CAG AGG3′
*Mast* (C)	Forward reverse	5′CAG CAT CGA GCA GAA GTA TAA GC3′ 5′TGG GTG GAG TTA TTG CAG TAG3′
Calreticulin (D1)	Forward reverse	5′GGA AAC CAC GTC AAA TTG 3′ 5′-GGT GAT GAG GAA ATT GTC-3′
*Spink2* Variant 2 (D2)	Forward reverse	5′GGC TAC TTG ACC ACT GC3′ 5′TTT GAG AAT CGG AAG AGT C3′
*Spink2* Variant 3 (D3)	Forward reverse	5′TTC CGA ACA CCA GAC TG3′ 5′ATG GCT ACC GTC CTC C3′
*Gapdh*	Forward reverse	5′TGA AGT CGC AGG AGA CAA CCT3′ 5′ATG GCC TTC CGT GTT CCT A3′

PCR conditions included an initial denaturation for 5 min at 95 °C followed by 45 cycles of denaturation at 95 °C for 10 s, annealing at 58 °C for 20 s and elongation at 72 °C for 30 s. The amplification of specific product was confirmed by melting curve profile. The relative fold change in expression between XY^RIII^ and XY^RIII^qdel mice was estimated based on Livak method (2^−ΔΔCt^).

### Small RNA isolation

Total RNA was extracted from tissues of XY^RIII^ and XY^RIII^qdel mice using TRIZOL reagent (Thermo Fisher Cat # 15596026). Total RNA was denatured at 65 °C for 10 min, incubated with 10% PEG-8000 (Sigma-Aldrich Cat # 25322-68-3) and 5 M NaCl for 30 min on ice and centrifuged at 7000 rpm for 7–10 min. The supernatant containing small RNA was precipitated overnight with 3 volumes of absolute alcohol and centrifuged at 13,000 rpm for 30 min. The small RNA pellet was washed with 80% ethanol and resuspended in RNase-free water. The quality of small RNA was checked on 12% Urea PAGE and quantitated using Nanodrop V-1000 (Thermo Fisher Scientific).

### Small RNA northern blotting

In total, 20–50 μg of small RNA from each tissue was resolved on a 12% Urea PAGE in 0.5× TBE running buffer and transferred onto Hybond N^+^ membrane. Decade marker (Thermo Fisher Cat # AM7778) was labelled and loaded according to the manufacturer’s instructions. In total, 10–25 μM of each LNA (locked nucleic acid) oligonucleotide probe (Exiqon) was end labelled for use as probes. Blots were hybridized (hybridization buffer—5× SSC, 5× Denhardt’s and 1% SDS) at 37 °C for 16–18 h and washed from 37 °C to 65 °C in 2× SSC, 0.2% SDS depending on the intensity of the signal. U6 was used as the loading control. Additional file 13: Fig. S6 shows the location of the LNA probes used for small RNA northern blots on the corresponding *Pirmy/Pirmy*-like RNAs.

### Electrophoretic mobility shift assay

RNA oligonucleotides corresponding to GAAGCAGAUGAGUAUAUG from *Sod* and UCAUUGGACAUAAACUGAAUUUUCCA from the gene for hypothetical protein spot A (Q9DAR0) were end labelled with γ-^32^P ATP and column purified using G-25 Sephadex (Sigma-Aldrich Cat # G2580-10G) and quantitated on a scintillation counter. EMSA (Electrophoretic mobility shift assay) reactions were set up in a total volume of 25 μl using binding buffer (20 mM HEPES, 3 mM MgCl2, 40 mM KCl, 5% glycerol, 2 mM DTT and 4 U of RNase inhibitor), with MIWI protein (5 μg per reaction). MIWI was overexpressed from a recombinant construct in pAAV-IRES-hrGFP vector and purified using the FLAG tag. Competitors, i.e. unlabeled oligonucelotides (30× concentration of hot oligo), MIWI (G82) antibody (90 ng, Cell Signaling Technology, Cat # 2079S) or Argonaute 3 antibody (100 ng, Abcam, Cat # ab3593), were added to the reaction, incubated for 1 h on ice, before addition of radio-labelled oligonucleotide (7000-10,000 cpm), and the entire mix was incubated on ice for another 30 min. EMSA was done on 5% native PAGE and image captured using FUJI phosphor Imager (FUJIFILM FLA-3000). A known piRNA, piR1, was used as the positive control, and Argonaute 3 antibody served as the antibody control.

### Luciferase assay

The 3′UTR sequences from *Sod, PLA2G12B* and *Cdc2l1* genes were amplified from mouse cDNA and cloned into the Luciferase pSP-luc + NF Fusion vector (Promega Cat # E4471). Either Luciferase gene alone or luciferase along with the UTR was cloned into pcDNA3.1 expression vector (Invitrogen, Cat # V790-20) for assaying the effect of antagopirs (Fig. 6G) on Luciferase expression. Co-transfection experiments were done using the GC-1spg cell line (ATCC CRL-2053) and lipofectamine 2000 (Thermo Fisher Scientific Cat # 11668019) using protocols specified by the manufacturer. Cells were seeded in 48-well plates, 24 h prior to transfection to obtain approximately 80% confluency. Each well was transfected with 50 ng of pcDNA3.1 plasmid containing either Luciferase gene alone or along with the cloned UTRs, 50 ng of the β-gal plasmid and varying concentrations of oligonucleotides complementary to the piRNA along with 0.5 μl of lipofectamine 2000 in antibiotic and serum-free DMEM (GIBCO). The complementary oligonucleotide to the piRNA has been designated as antagopirs. The antagopirs (*Sod* - 5′GAAGCAGAUGAGUAUAUG3′; *PLA2G12B* - 5′CCAAACUGUUGGAAGAAGGAAU3′) were procured as RNA oligonucleotides from Eurofins Genomics India Pvt. Ltd, Bangalore, India. Different concentrations of antagopirs (0 nM, 5 nM, 10 nM and 20 nM / well) were tested in the assay for their effect on the UTRs (Fig. 6H). Five hours post transfection, the medium was replaced with a complete growth medium. The cell extracts were prepared 24 h post transfection using Reporter Lysis Buffer (Promega Cat # E4030) and assayed for Luciferase activity in EnSpire 2300 multimode plate reader (Perkin Elmer). The Luciferase activity was normalized using β galactosidase. Three independent sets of experiments were done in triplicate. Values of Mean ± SEM (*n* = 3) were calculated. Statistical analysis was performed using an unpaired *t*-test to calculate the level of significance.

## Supplementary Information

**Additional file 1: Figure S1.** Developmental stage-specific expression of M34 (DQ907163). Testis sections from 1 month old, new born and 18.5d embryo showing expression of M34 from 18.5d embryonic stage onwards. The signals localize to the nucleus in 18.5d embryos and translocate to the cytoplasm at later stages. Arrows indicate cells surrounding the testicular cords (TC). Left hand side panels show the merged images from FITC-tagged probe and the nuclei counterstained with propidium iodide. Middle panels show signals from the FITC-tagged M34 alone. Right hand side panels counterstained with propidium iodide show the testicular histology at different developmental stages.

**Additional file 2: Figure 2A.** Raw data relating to Figure 2A. Full picture showing the reverse transcription PCR samples from XY^RIII^ brain and testis and XY^RIII^qdel testis separated in 1.5% agarose gel. The lane labeled –ve is the negative control without sample cDNA. The gel picture is cropped to show only XY^RIII^ brain and testis samples in Fig. 2A.

**Additional file 3: Figure S2.** The spectrum of splicing–patterns present in *Pirmy* splice isoforms. Analysis of the splicing patterns in *Pirmy* splice isoform sequences showed different patterns of splicing like A exon skipping B alternative 5’ splice sites, C alternative 3’ splice sites, D mutually exclusive alternative exons, E intron retention and F complex events. Left panel highlights the region of splicing in the isoforms encircled in black.

**Additional file 4: Supplementary data sheet.** Annotation of the splice junctions of *Pirmy* isoforms with reference to genomic contig NT_166343.2: The exon-intron junctions of all 80 splice isoforms have been annotated by aligning them to the genomic contig NT_166343.2. The splice junctions of all the *Pirmy* isoforms show the consensus splice signal sequences AG/GT, which have been represented with green and blue color respectively. The 5’ and 3’ flanking sequences of all the splice junctions are given (black letters).

**Additional file 5: Figure S3.** Figure showing reduced transcription of the genomic clone M34 and its subclones. A Shows the reduction in signal elicited by M34 clone on a metaphase spread, testis and sperms of XY^RIII^qdel mice when compared to the wild type, XY^RIII^. Sperms collected from epididymis show very low levels of RNA compared to sperms from testis. There is no marked difference in fluorescence intensity between XY^RIII^ and XY^RIII^qdel sperms collected from epididymis. B Shows localization of the subclones p17, p21, p32 and p66 onto the parental clone M34 (DQ907163) and the sequencing strategy used to cover the entire M34 clone C This panel shows clear reduction in expression of different subclones p17, p21, p32 and p66 of M34 in testis sections from XY^RIII^qdel mice. Green fluorescence represents signal from FITC labelled probe, nuclei are counterstained with propidium iodide (red). Yellow indicates co-localization.

**Additional file 6: Figure S4.** Genomic Copy number analysis of *Pirmy* and *Pirmy-*like RNAs in genomes of XY^RIII^ and XY^RIII^qdel mice. This figure shows the relative copy number difference of *Pirmy* and *Pirmy*-like genes (exon 7 of DQ907162) in the genomes of XY^RIII^ and XY^RIII^qdel mice. Values represent mean ± SEM. Statistical analysis was performed using an unpaired t-test and the significance level is represented as ** *P* < 0.01. XY^RIII^qdel mice showed a 3 fold reduction in genomic copy number of *Pirmy* and *Pirmy-*like RNAs compared to XY^RIII^.

**Additional file 7:** qPCR raw data relating to Figure S4. Excel sheet showing the Ct values from qPCR and the calculations for estimation of copy number of *Pirmy* and *Pirmy-*like RNAs in XY^RIII^ and XY^RIII^qdel genomes.

**Additional file 8: Movie S1.** Motility pattern of XY^RIII^ spermatozoa. shows the motility pattern of spermatozoa from XY^RIII^ mice. Movie S1 shows that most of the sperms are motile in XY^RIII^. They show progressive motility as moving spermatozoa cover a distance as they move.

**Additional file 9: Movie S2.** Aberrant motility pattern of XY^RIII^qdel spermatozoa. This movie shows the motility pattern of XY^RIII^qdel spermatozoa. These mice have fewer motile sperms and aberrant motility patterns. Most of the motile sperms move at the same position often in circles, and do not cover any distance as they move.

**Additional file 10: Figure S5.** Comparative quantification of deregulated proteins and genes in XY^RIII^ and XY^RIII^qdel mice. A Graph represents the mean intensity (arbitrary units) observed on comparing five sets of gels after normalization against control spots (equal expression), from XY^RIII^ and XY^RIII^qdel sperm proteome for the differentially expressed protein spots in the pI range of 4-7. Error bars represent the standard deviation. B Western blotting to confirm the expression levels of two proteins (D4 and D5) identified on 2D-gels; differential expression is observed in sperms (RT - XY^RIII^ testis, YT - XY^RIII^qdel testis, RS - XY^RIII^ sperm, YS - XY^RIII^qdel sperm). The lower sub-panel for all blots is the loading control using Tubulin. C represents the relative expression level of different testis genes in XY^RIII^qdel in comparison with XY^RIII^, performed using qPCR. Values represent mean ± SEM (n = 6). Statistical analysis was performed using an unpaired t-test to compare each gene between the two groups and the level of significance is represented as ** P < 0.01; * P < 0.05. Significant increase in expression was observed in Calreticulin (P = 0.006), *Sdf2l1* (P = 0.002), *Mast* (P = 0.04) and *Spink2* variant 2 (P = 0.01) in XY^RIII^qdel compared to XY^RIII^ mice. Meanwhile, no change in expression was observed in spot A Riken cDNA and *Spink2* variant 3 between both the groups. D Northern blot analysis of *Sod* and *Fabp9* in brain, testis and ovary. Both the genes show upregulated expression in XY^RIII^qdel testis compared to XY^RIII^. There is no detectable expression in brain and ovary. (RB- XY^RIII^ brain, YB- XY^RIII^qdel brain; RT- XY^RIII^ testis, YT- XY^RIII^qdel testis; FB- Female brain, Ov- ovary, M- marker).

**Additional file 11:** Raw data relating to **Figure S5B.** This figure shows scans of the original Western blots of two sets each of SOD and FABP9.

**Additional file 12:** Raw data relating to **Figure S5C.** Excel sheet showing the Ct values from qPCR and the calculations for transcript expression levels in testes from XY^RIII^ and XY^RIII^qdel mice.

**Additional file 13: Figure S6.** Localization of probes to the *Pirmy* and *Pirmy*-like RNAs. Positions of the small RNA probes from *Pirmy* and *Pirmy*-like RNAs used for northern blotting and the names of the genes with homology to *Pirmy* and *Pirmy*-like RNAs are marked in red. Antisense probes are shown in purple with the corresponding gene names on the left. The LNA oligonucleotides used as antagopirs are indicated on the right-hand side of corresponding sequences.

**Additional file 14: Figure S7.** Small RNA Northern blots using piRNA probes with different tissues from XY^RIII^ and XY^RIII^qdel. Small RNA northern blots showing expression of piRNAs from both XY^RIII^ and XY^RIII^qdel testis, using stretches homologous to *Pirmy* splice variants and *Pirmy*-like RNAs in the UTRs of Spot A (ProtA1, ProtA2, ProtA3), *Sod, Bche, PLA2G12B, Mads* and *Oosp1.*No significant difference is observed in piRNA signals (indicated by arrows) between XY^RIII^ and XY^RIII^qdel testis. The genes and the corresponding ncRNAs are as indicated in the blots. The lower panel corresponds to signal from U6 used as loading control

**Additional file 15: ** Raw data relating to **Figure 6H.** Sheets 1 and 2 contain the raw data for SOD and PLA2G12B along with the corresponding control used; Sheet 3 shows the computations for drawing the graph.

**Additional file 16: Table S1.** piRNA mapping to *Pirmy* and *Pirmy*-like RNAs in SRA database. Table showing piRNA sequences from *Pirmy and Pirmy-like RNAs* aligning to piRNAs in SRA databases (SRP000623 and SRP001701). The chromosomal localization and copy number of these piRNAs are also given in the table.

**Additional file 17: Figure S8.** Localization of deregulated proteins to mouse sperm. Figure shows the localization of SPIKN2, FABP9, Acrosin Trypsin Inhibitor, Calreticulin, SOD and MAST proteins onto mouse sperm. The function of each of these proteins is indicated.

**Additional file 18: Table S2.** The genes that are upregulated in XY^RIII^q-del mice with sequence homology to *Pirmy* and *Pirmy*-like RNAs in their UTRs. Table shows the list of upregulated genes in XY^RIII^q-del mice testis [53] that show homology to *Pirmy* and *Pirmy*-like RNAs in their 3’/5’UTRs, chromosomal localization and expression pattern of these genes in the above study.

## Data Availability

All data generated or analysed during this study are included in this published article, its supplementary information files, and publicly available repositories. All sequences from this study have been deposited in NCBI database. With the accession numbers: *Pirmy* DQ907162, *Pirmy* splice isoforms FJ541103- FJ541181, *Pirmy*-like RNAs FJ541075-FJ541102. Novel proteins identified in the study have been deposited in the Uniprot database as Q9DAR0 (A), Q7TPM5 (C) and Q8BMY7 (Spink2 variant 3). Sequence Read Archives SRP001701 and SRP000623 were used for analysing the ncRNAs. Raw data can be found in Additional files 4, 7, 12 and 15.

## Abbreviations

MSYq: Male-specific region on Yq
Yq: Long arm of Y chromosome
XY^RIII^qdel: Mice with 2/3 interstitial deletion of the Y long arm of XY^RIII^ strain
UTR: Untranslated region
piRNA: PIWI interacting RNA
*Pirmy*: piRNA from mouse Y chromosome
lnc RNA: Long noncoding RNA
FISH: Fluorescence in situ hybridization
EST: Expressed Sequence Tags
RT-PCR: Reverse transcription PCR
EMSA: Electrophoretic mobility shift assay
LNA: Locked nucleic acids
PIWI: P-element-induced wimpy testis
MIWI: Mouse homologue of PIWI
