# Supplementary figures and images for: Y chromosomal noncoding RNAs regulate autosomal gene expression via piRNAs in mouse testis
Source: BMC Biol. 2021 Sep 9;19:198. doi: 10.1186/s12915-021-01125-x (PMC8428117; doi:10.1186/s12915-021-01125-x)

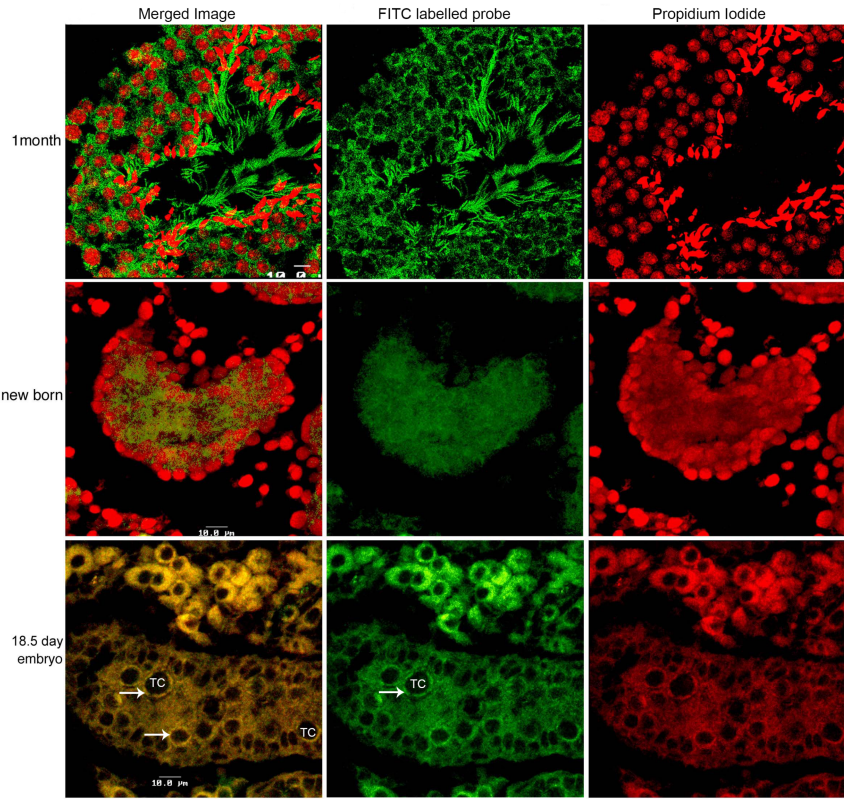

Supplement: Supplementary file 1 — Additional file 1: Figure S1. Developmental stage-specific expression of M34 (DQ907163). Testis sections from 1 month old, new born and 18.5d embryo showing expression of M34 from 18.5d embryonic stage onwards. The signals localize to the nucleus in 18.5d embryos and translocate to the cytoplasm at later stages. Arrows indicate cells surrounding the testicular cords (TC). Left hand side panels show the merged images from FITC-tagged probe and the nuclei counterstained with propidium iodide. Middle panels show signals from the FITC-tagged M34 alone. Right hand side panels counterstained with propidium iodide show the testicular histology at different developmental stages. [file 12915_2021_1125_MOESM1_ESM.pdf]

100bp  
ladder

XY<sup>RIII</sup>  
Brain

XY<sup>RIII</sup>  
Testis

XY<sup>RIII</sup>qdel  
Testis

-ve

1500  
1200  
1000

500

200

100

1000

500

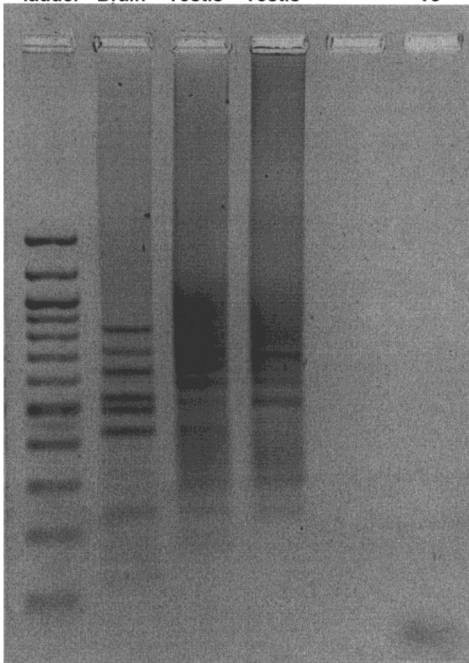

Supplement: Supplementary file 2 — Additional file 2: Figure 2A. Raw data relating to Figure 2A. Full picture showing the reverse transcription PCR samples from XYRIII brain and testis and XYRIIIqdel testis separated in 1.5% agarose gel. The lane labeled –ve is the negative control without sample cDNA. The gel picture is cropped to show only XYRIII brain and testis samples in Fig. 2A. [file 12915_2021_1125_MOESM2_ESM.pdf]

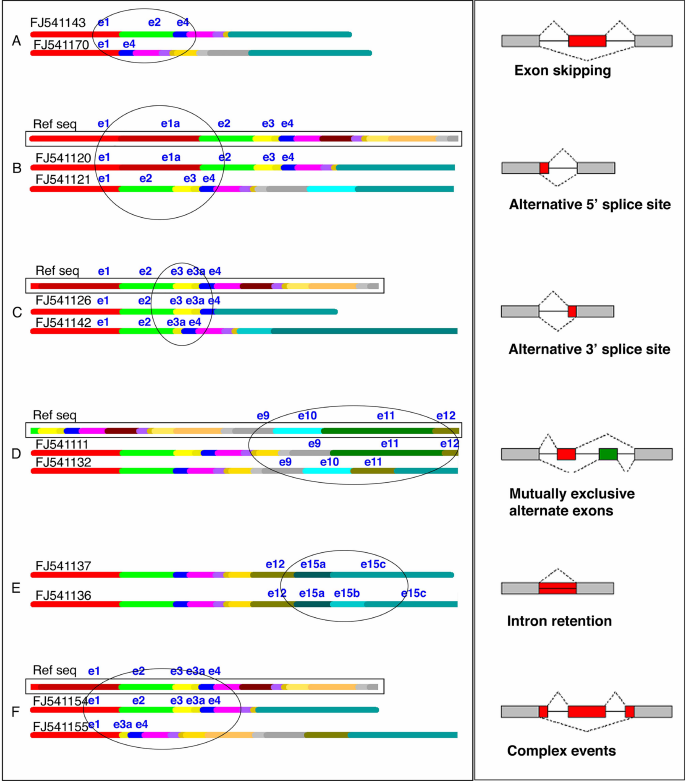

Supplement: Supplementary file 3 — Additional file 3: Figure S2. The spectrum of splicing–patterns present in Pirmy splice isoforms. Analysis of the splicing patterns in Pirmy splice isoform sequences showed different patterns of splicing like A exon skipping B alternative 5’ splice sites, C alternative 3’ splice sites, D mutually exclusive alternative exons, E intron retention and F complex events. Left panel highlights the region of splicing in the isoforms encircled in black. [file 12915_2021_1125_MOESM3_ESM.pdf]

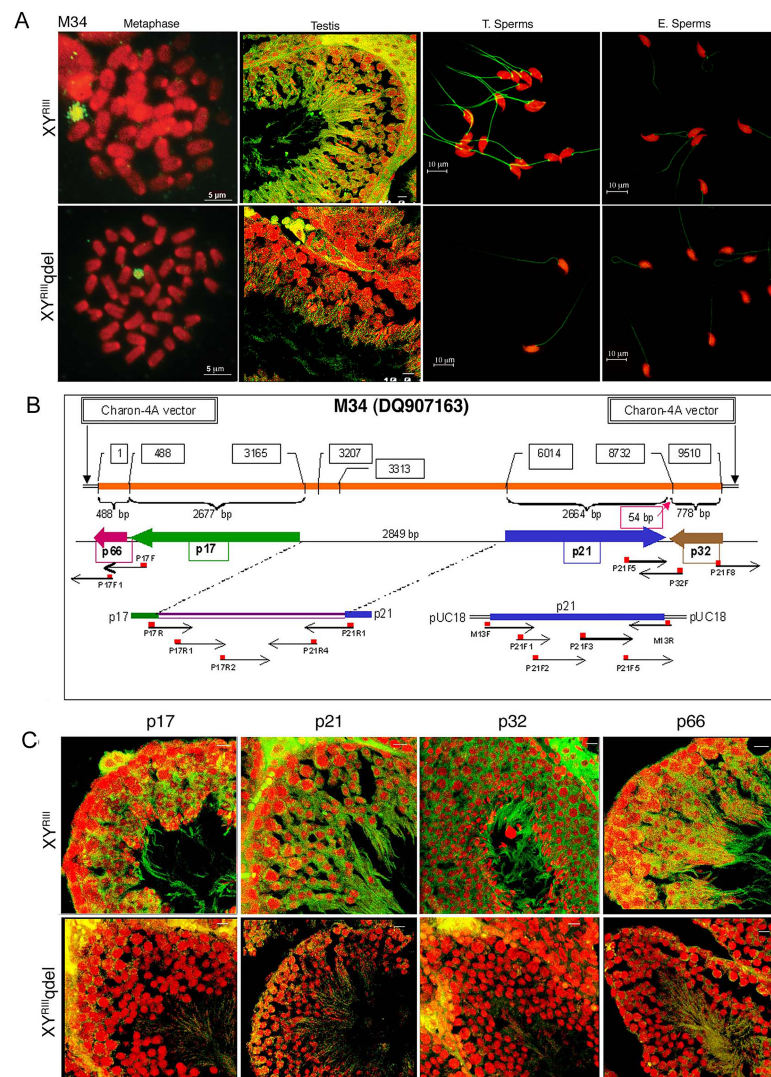

Supplement: Supplementary file 5 — Additional file 5: Figure S3. Figure showing reduced transcription of the genomic clone M34 and its subclones. A Shows the reduction in signal elicited by M34 clone on a metaphase spread, testis and sperms of XYRIIIqdel mice when compared to the wild type, XYRIII. Sperms collected from epididymis show very low levels of RNA compared to sperms from testis. There is no marked difference in fluorescence intensity between XYRIII and XYRIIIqdel sperms collected from epididymis. B Shows localization of the subclones p17, p21, p32 and p66 onto the parental clone M34 (DQ907163) and the sequencing strategy used to cover the entire M34 clone C This panel shows clear reduction in expression of different subclones p17, p21, p32 and p66 of M34 in testis sections from XYRIIIqdel mice. Green fluorescence represents signal from FITC labelled probe, nuclei are counterstained with propidium iodide (red). Yellow indicates co-localization. [file 12915_2021_1125_MOESM5_ESM.pdf]

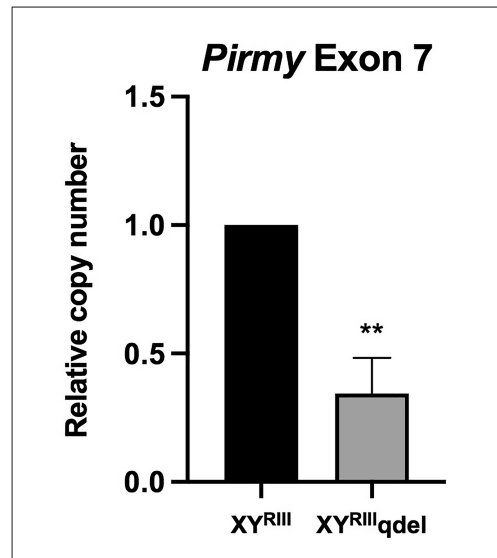

Supplement: Supplementary file 6 — Additional file 6: Figure S4. Genomic Copy number analysis of Pirmy and Pirmy-like RNAs in genomes of XYRIII and XYRIIIqdel mice. This figure shows the relative copy number difference of Pirmy and Pirmy-like genes (exon 7 of DQ907162) in the genomes of XYRIII and XYRIIIqdel mice. Values represent mean ± SEM. Statistical analysis was performed using an unpaired t-test and the significance level is represented as ** P < 0.01. XYRIIIqdel mice showed a 3 fold reduction in genomic copy number of Pirmy and Pirmy-like RNAs compared to XYRIII. [file 12915_2021_1125_MOESM6_ESM.pdf]

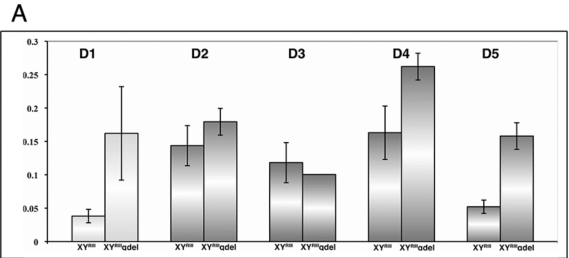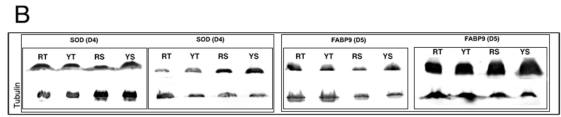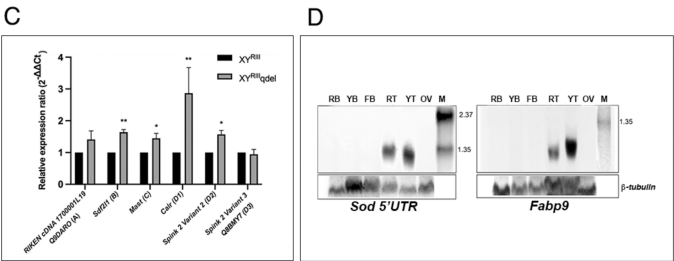

Supplement: Supplementary file 10 — Additional file 10: Figure S5. Comparative quantification of deregulated proteins and genes in XYRIII and XYRIIIqdel mice. A Graph represents the mean intensity (arbitrary units) observed on comparing five sets of gels after normalization against control spots (equal expression), from XYRIII and XYRIIIqdel sperm proteome for the differentially expressed protein spots in the pI range of 4-7. Error bars represent the standard deviation. B Western blotting to confirm the expression levels of two proteins (D4 and D5) identified on 2D-gels; differential expression is observed in sperms (RT - XYRIII testis, YT - XYRIIIqdel testis, RS - XYRIII sperm, YS - XYRIIIqdel sperm). The lower sub-panel for all blots is the loading control using Tubulin. C represents the relative expression level of different testis genes in XYRIIIqdel in comparison with XYRIII, performed using qPCR. Values represent mean ± SEM (n = 6). Statistical analysis was performed using an unpaired t-test to compare each gene between the two groups and the level of significance is represented as ** P < 0.01; * P < 0.05. Significant increase in expression was observed in Calreticulin (P = 0.006), Sdf2l1 (P = 0.002), Mast (P = 0.04) and Spink2 variant 2 (P = 0.01) in XYRIIIqdel compared to XYRIII mice. Meanwhile, no change in expression was observed in spot A Riken cDNA and Spink2 variant 3 between both the groups. D Northern blot analysis of Sod and Fabp9 in brain, testis and ovary. Both the genes show upregulated expression in XYRIIIqdel testis compared to XYRIII. There is no detectable expression in brain and ovary. (RB- XYRIII brain, YB- XYRIIIqdel brain; RT- XYRIII testis, YT- XYRIIIqdel testis; FB- Female brain, Ov- ovary, M- marker). [file 12915_2021_1125_MOESM10_ESM.pdf]

**SOD-Set 1**

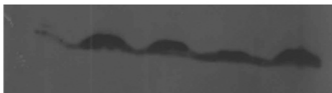

**SOD-Set 2**

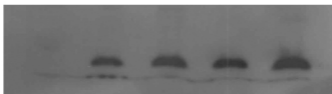

**FABP9-Set 1**

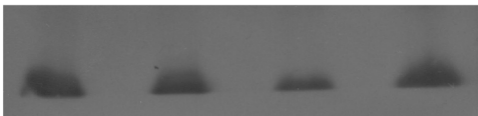

**FABP9-Set 2**

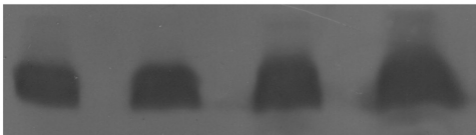

Supplement: Supplementary file 11 — Additional file 11: Raw data relating to Figure S5B. This figure shows scans of the original Western blots of two sets each of SOD and FABP9. [file 12915_2021_1125_MOESM11_ESM.pdf]

FJ541118.1 (599bp)

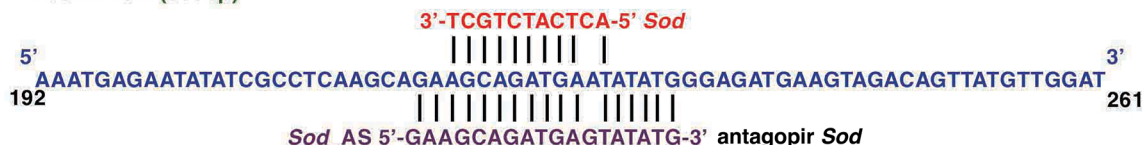

FJ541161.1 (260bp)

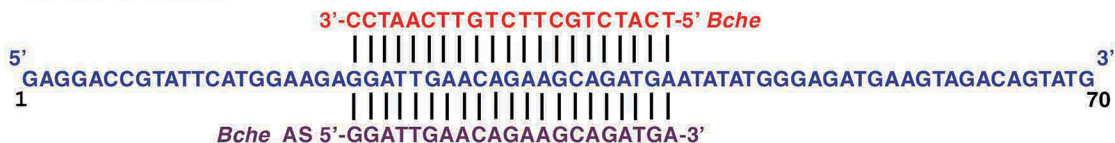

FJ541135.1 (808bp)

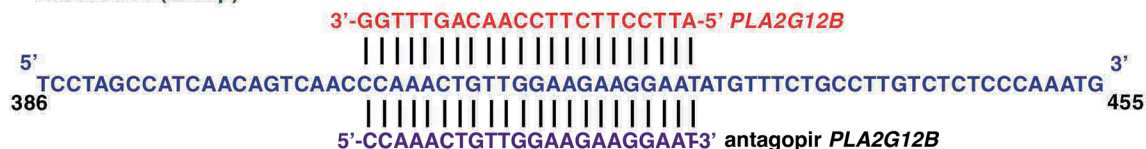

FJ541077.1 (752bp)

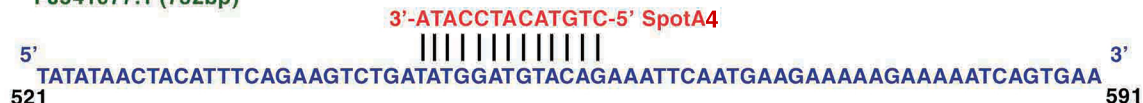

FJ541087.1 (821bp)

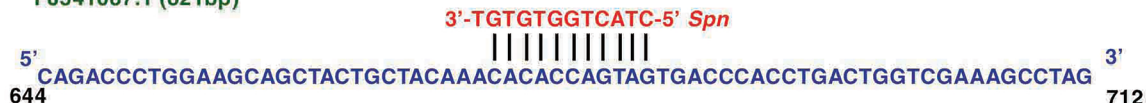

FJ541091.1 (203bp)

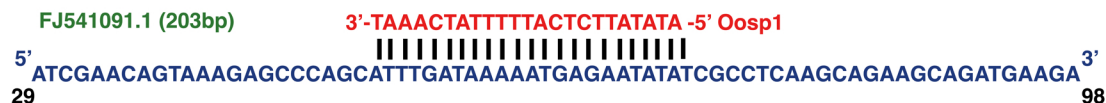

FJ541091.1 (203bp)

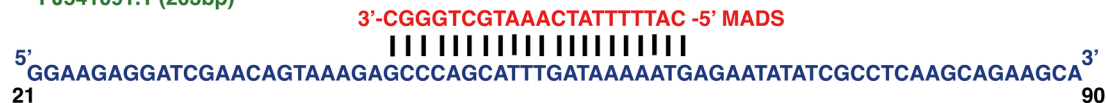

FJ541077.1 (752bp)

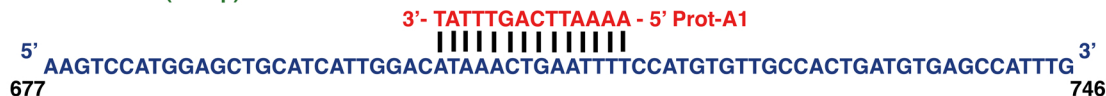

FJ541083.1 (594bp)

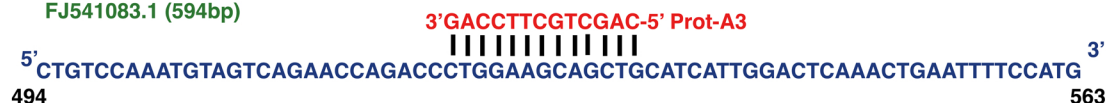

Supplement: Supplementary file 13 — Additional file 13: Figure S6. Localization of probes to the Pirmy and Pirmy-like RNAs. Positions of the small RNA probes from Pirmy and Pirmy-like RNAs used for northern blotting and the names of the genes with homology to Pirmy and Pirmy-like RNAs are marked in red. Antisense probes are shown in purple with the corresponding gene names on the left. The LNA oligonucleotides used as antagopirs are indicated on the right-hand side of corresponding sequences. [file 12915_2021_1125_MOESM13_ESM.pdf]

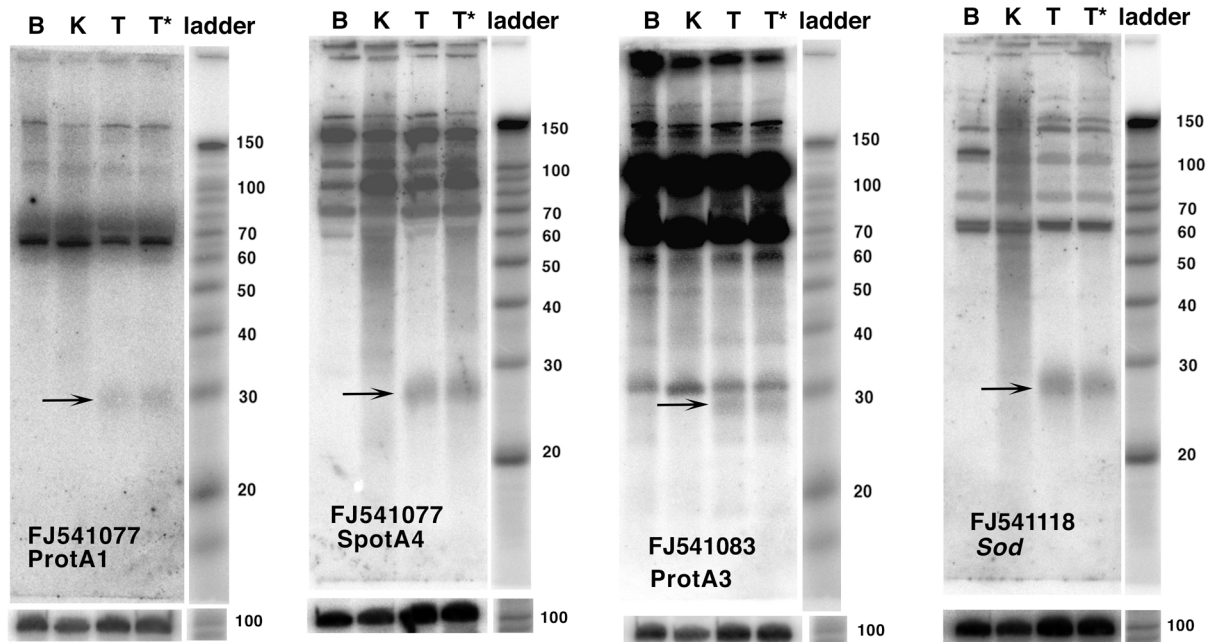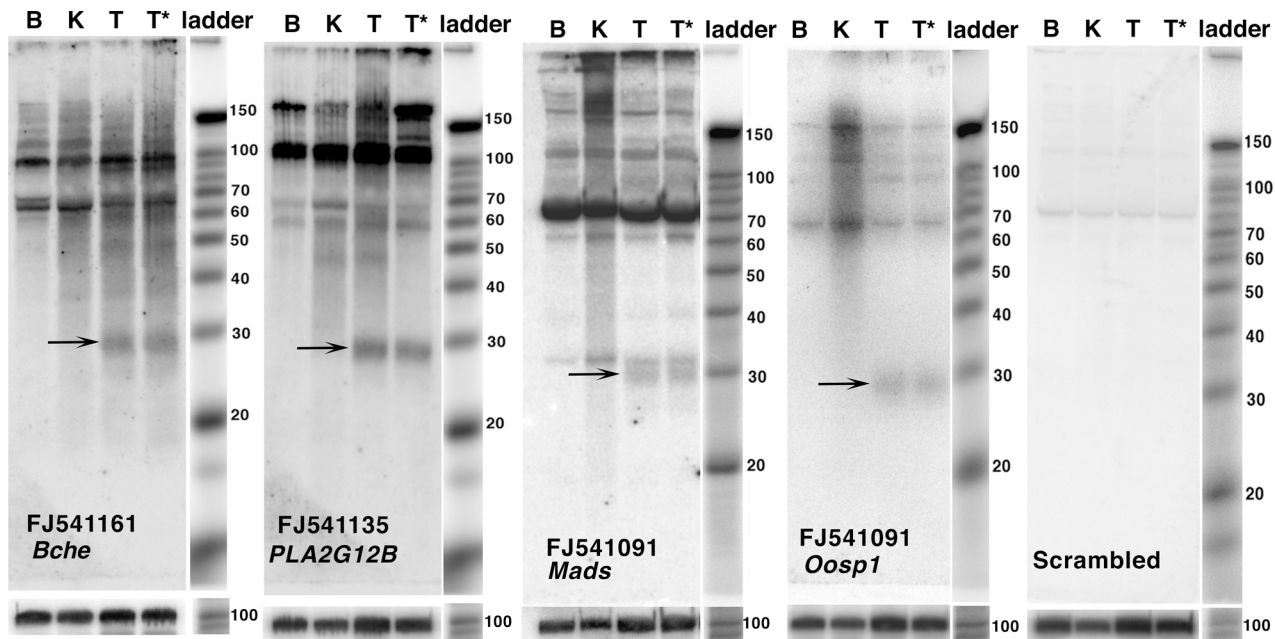

Supplement: Supplementary file 14 — Additional file 14: Figure S7. Small RNA Northern blots using piRNA probes with different tissues from XYRIII and XYRIIIqdel. Small RNA northern blots showing expression of piRNAs from both XYRIII and XYRIIIqdel testis, using stretches homologous to Pirmy splice variants and Pirmy-like RNAs in the UTRs of Spot A (ProtA1, ProtA2, ProtA3), Sod, Bche, PLA2G12B, Mads and Oosp1.No significant difference is observed in piRNA signals (indicated by arrows) between XYRIII and XYRIIIqdel testis. The genes and the corresponding ncRNAs are as indicated in the blots. The lower panel corresponds to signal from U6 used as loading control [file 12915_2021_1125_MOESM14_ESM.pdf]

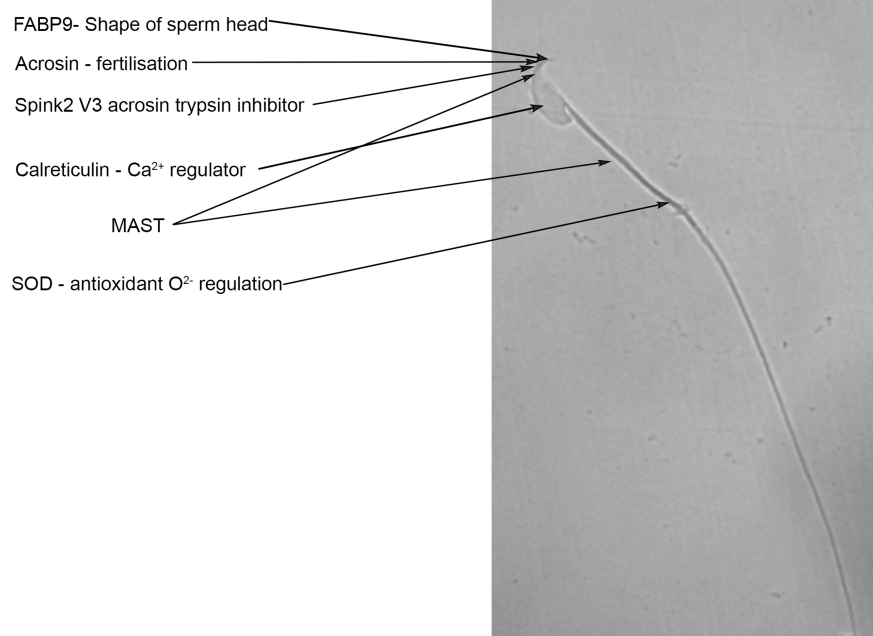

Supplement: Supplementary file 17 — Additional file 17: Figure S8. Localization of deregulated proteins to mouse sperm. Figure shows the localization of SPIKN2, FABP9, Acrosin Trypsin Inhibitor, Calreticulin, SOD and MAST proteins onto mouse sperm. The function of each of these proteins is indicated. [file 12915_2021_1125_MOESM17_ESM.pdf]
