# Supplementary material for: Y chromosomal noncoding RNAs regulate autosomal gene expression via piRNAs in mouse testis
Source: BMC Biol. 2021 Sep 9;19:198. doi: 10.1186/s12915-021-01125-x (PMC8428117; doi:10.1186/s12915-021-01125-x)
Supplement: Supplementary file 18 — Additional file 18: Table S2. The genes that are upregulated in XYRIIIq-del mice with sequence homology to Pirmy and Pirmy-like RNAs in their UTRs. Table shows the list of upregulated genes in XYRIIIq-del mice testis [53] that show homology to Pirmy and Pirmy-like RNAs in their 3’/5’UTRs, chromosomal localization and expression pattern of these genes in the above study. [file 12915_2021_1125_MOESM18_ESM.docx]

**Table S2.** The genes that are upregulated in XY^RIII^q-del mice (Ellis et al. 2005) with sequence homology to *Pirmy* and *Pirmy*-like RNAs in their UTRs.

| **S. No.** | **Gene name**  **(Accession No.)** | **Chromosome**  **localization** | **Expression Pattern** | **UTR Homology** |
| --- | --- | --- | --- | --- |
|  |  |  |  |  |
| 1 | Riken cDNA (AK006251) | X | T/U | 3’ UTR |
| 2 | Xlr-related, meiosis regulated (Xmr) (NM_009529) | X | T | 3’ UTR  5’UTR |
| 3 | Tgif2lX1, TGFB induced actor homeobox 2-like, x-linked 1 (NM_153109) | X | T | 3’ UTR |
| 4 | Riken cDNA (AK005817) | X | T | 3’ UTR |
| 5 | Uncharacterized protein (NM_198677) | 14 | T | 3’ UTR |
| 6 | Tspan6, tetraspanin 6 (NM_019656) | X | T/U | 3’ UTR |
| 7 | Riken cDNA, contains lysine-rich region (AK006132) | 12 | T | 3’ UTR |
| 8 | Cyst9 (NM_009979) | 2 | T | 5’ UTR |
| 9 | Grhpr (NM_080289) | 4 | T/U | 3’ UTR |
| 10 | PWWP domain containing 2B (AK086357) | 7 | T/U | 3’ UTR |

Table shows the list of upregulated genes in XY^RIII^q-del mice testis that show homology to *Pirmy* and *Pirmy*-like RNAs in their 3’/5’UTRs, chromosomal localization of these genes in the above study.
